# Neurotoxicity of diesel exhaust extracts in zebrafish and its implications for neurodegenerative disease

**DOI:** 10.1038/s41598-022-23485-2

**Published:** 2022-11-12

**Authors:** Sung Min Ha, Lisa M. Barnhill, Sharon Li, Jeff M. Bronstein

**Affiliations:** 1 Department of Integrative Biology and Physiology, David Geffen School of Medicine at UCLA, 710 Westwood Plaza, Los Angeles, CA 90095 USA; 2 Department of Neurology, David Geffen School of Medicine at UCLA, 710 Westwood Plaza, Los Angeles, CA 90095 USA; 3 Department of Molecular Toxicology, David Geffen School of Medicine at UCLA, 710 Westwood Plaza, Los Angeles, CA 90095 USA

**Keywords:** Diseases of the nervous system, Glial biology

## Abstract

Long-term air pollution (AP) exposure, including diesel exhaust exposure, is increasingly being recognized as a major contributor to the development of neurodegenerative diseases such as Parkinson’s and Alzheimer’s disease. How AP increases the risk of neurodegeneration is not well understood but might include direct neurotoxicity and CNS inflammation. We investigated the impact of diesel exhaust particulate extract (DEPe) exposure on the brain and the mechanisms by which microglia and astroglia might mediate neuronal changes. Zebrafish (ZF) were utilized to determine neuronal toxicity of and microglial response to DEPe and single cell RNA sequencing was employed to study cell type-specific transcriptomic responses within the ZF brain. DEPe exposure induced neuronal injury and microglial activation in vivo. However, preventing the development of microglia did not attenuate DEPe-induced neuron loss, leading us to investigate microglial, astroglial, and neuronal response to DEPe exposure at single-cell resolution. Differentially expressed genes and disease-relevant pathways were identified within glial and neuronal clusters after DEPe exposure. Microglia and astroglia existed in multiple states, some of which appear toxic and others protective to neurons. Neuronal transcriptomic analysis revealed that DEPe exposure reduced expression of autophagy-related genes consistent with direct neurotoxicity. In summary, DEPe exposure was neurotoxic in developing ZF larvae and induced neuroinflammation. The microglial inflammatory response did not contribute to neurotoxicity of DEPe and in fact, some glial clusters upregulated transcriptional pathways that are likely protective. Furthermore, DEPe exposure led to reduced expression of autophagy-related genes in neurons that likely contribute to its toxicity.

## Introduction

According to data available in 2017, 92% of the world’s population lived in areas that did not meet WHO guidelines for air quality^1^. Although the links between air pollution exposure and cardiovascular and respiratory health are well-established, the role that decades of air pollution exposure plays in the development of neurodegenerative disease has only recently been recognized. A growing number of epidemiological studies have established links between exposure to traffic-related air pollution and the development of various neurodegenerative diseases, including Alzheimer’s disease (AD) and Parkinson’s Disease (PD)^2–4^. As the median age of the global population increases and air pollution remains the world’s largest environmental health risk, it is vital to determine if AP exposure increases the risk of developing neurodegenerative disease and if so, what are the mechanisms by which it acts.

Establishing biological plausibility is necessary before concluding that an association of an environmental insult such as AP is causally linked to increased risk of disease. Neurodegenerative diseases are generally defined by neuronal dysfunction and death. In addition, the accumulation of aggregated and misfolded proteins is widely found in brains of patients with PD and AD. α-Synuclein forms intracytoplasmic inclusions called Lewy bodies in PD and Abeta amyloid and tau form plaques and tangles in AD. Evidence of neuroinflammation (e.g. activation of microglia and astrocytes) has been widely observed both in patients and animal models of AD and PD^5,6^. The role of microglia and astrocytes in the development and progression of neurodegenerative diseases is likely complex, especially when considering the effects of environmental exposures such as AP. Both protective and detrimental effects of neuroinflammation have been reported in response to environmental stressors and in neurodegenerative disease, but little is known about the glial response to AP. There are few animal studies investigating the effects of AP on the brain, but some reports have described evidence of neuronal loss and inflammation when exposed to components of AP (e.g. diesel exhaust)^7–9^.

Recently, we found that ZF (*Danio rerio*) exposed to DEPe, resulted in neuronal loss, accumulation of ɣ1 synuclein (the ZF equivalent to mammalian α-synuclein), and inhibition of autophagic flux^9^. ZF are a vertebrate model organism well-suited to study the neurotoxicity of environmental chemicals due to their small size, rapid development, and transparency so florescent reporters can be imaged in living fish. In mammals, DE is inhaled and many of the components of DE pass into the brain via the blood stream or directly through the olfactory system. Under natural conditions, ZF are exposed only to the components of air that are dissolved in the water. In this report, we focused on the mechanisms of neurotoxicity of the components of DE that are dissolved in the water and make it to the brain using concentrations equivalent to those found in human brain tissue.

## Materials and methods

### Zebrafish husbandry

ZF were raised at 28.5 °C on a 14-h light, 10-h dark cycle. The lines used were AB (wild-type), Vmat2:GFP^10^, mpeg1*:*mCherry^11^, Tg(isl1[ss]:Gal4-VP16,UAS:eGFP)^zf154^^12^. All zebrafish lines were used in accordance with the UCLA Animal Research Council and Division of Laboratory Animal Medicine guidelines. The UCLA Division of Laboratory Animal Medicine (DLAM) oversees all vertebrate animal investigations and is an approved AALAC facility. This study is reported in accordance with ARRIVE guidelines.

### Zebrafish DEPe treatment

DEPe was prepared from National Institute of Standards and Technology (NIST) SRM #1975, a dichloromethane extract of diesel particulate matter. It was dried using nitrogen gas and the remaining particulate matter was resuspended in dimethyl sulfoxide (DMSO). The extract was submitted for further component analysis by Dr. James Schauer from the University of Wisconsin-Madison. The presence of 120 compounds was analyzed and final concentration calculated (Supplemental Table S1). Embryos for treatment were manually dechorionated at 24 h post-fertilization (hpf). Each treatment consisted of 20 embryos in a final volume of 10 mL E3 buffer. The final concentration of DEPe in the treatment well was 20 µg/ml. DMSO vehicle concentration was 0.1% in both treatments and controls. Embryos were treated until 3–5 days post-fertilization (dpf) and were euthanized using tricaine methanesulfonate (final concentration 0.5 mg/mL).

### Inhibition of microglial development

Reducing expression of PU.1 has been shown to inhibit macrophage (including microglia) development and can be accomplished using antisense morpholinos^13^. PU.1 morpholino oligonucleotides or scramble morpholino solutions (0.4 mM), were injected into homozygous *mpeg1:mCherry* embryos at the 1–4 cell stage. The embryos were treated at 24hpf with DMSO or DEPe as described above, until 3dpf.

### Zebrafish imaging

After the larvae were euthanized with tricane, they were fixed in 4% PFA for 4 h at 25 °C. They were washed with 1X Dulbecco’s Phosphate Buffered Saline (DPBS, Thermo Fisher Scientific, Waltham, MA) and permeabilized in a solution of 10 µg/mL Proteinase K for 5–7 min. The Proteinase K was washed off with ddH2O for 5 min. The larvae were placed in a 10% blocking solution of 5% BSA + 5% goat serum for 1 h. Primary antibody (anti-GFP antibody A11120 and anti-mCherry PA5-34974, ThermoFisher Scientific, Waltham, MA) was applied in 2% blocking solution of 1% BSA + 1% goat serum overnight at 4 °C. The antibody was washed 8 times for 15 min each, and the secondary antibody was applied in 2% blocking solution. After washing off the secondary antibody, the sample was placed in 50% glycerol for 30 min, and cleared in 100% glycerol until time of dissection. Larvae were dissected using Dumont #5 Fine Forceps. Brains were carefully removed and mounted in 100% glycerol for imaging on Leica SPE (Leica Microsystems Inc, Buffalo Grove, IL). Aminergic neurons in the telencephalon and diencephalon, microglia, and islet neurons were imaged in Z-stacks of 2um and analyzed as previously described^9^.

### Microglial analysis

Microglial activation was quantified using FIJI/ImageJ. Each image file was Z-projected, converted to grayscale (16-bit), brightness/contrast were adjusted to eliminate background. The images were then made binary, skeletonized, and the program Simple Neurite Tracer (https://imagej.net/plugins/snt/) was used while observing original Z-projected image to select microglial branches, and Analyze Skeleton was performed^14^.

### RNA extraction, cDNA preparation, and rtPCR analysis

5dpf DMSO or DEPe larvae were euthanized and RNA extraction was performed using TRIzol reagent (Invitrogen, Waltham, MA) according to product protocols. Collected RNA was converted to cDNA using iScript cDNA Synthesis Kit (Bio-Rad Laboratories, Hercules, CA). rtPCR reaction was conducted using SsoAdvanced Universal SYBR Green Supermix (Bio-Rad Laboratories), cDNA, and primers for the genes of interest (Supplemental Table S2). Cycle number data was analyzed to represent fold change values using the 2^-ddcT^ method.

### Lysotracker green application and live imaging

5dpf DMSO or DEPe larvae were incubated for 15 min at room temperature in the dark in 10uM Lysotracker Green (Thermo Fisher Scientific) diluted in E3 buffer. After incubation, the larvae were washed 3 times in fresh E3 buffer and placed into a 5 ml tricaine methanesulfonate (150ul 5 mg/ml tricaine methanesulfonate + 4.85 ml E3) solution for anesthesia. The larvae were mounted in 1% agarose and live-imaged using Leica SPE (Leica Microsystems Inc, Buffalo Grove, IL). The microglia and Lysotracker Green-labeled intracellular regions were quantified for intensity using FIJI/ImageJ.

### 10X Genomics scRNA-seq

pGFAP-GFP plasmid was injected into homozygous *mpeg1:mCherry* embryos at the 1–4 cell stage^15^. Thirty embryos per condition were treated at 24hpf with DMSO or DEPe and anesthetized with tricaine methanesulfonate at 5dpf. The head tissue, with eyes removed, was placed in 1 ml cold Ringer’s Solution on ice and pelleted in 1.5-ml microcentrifuge tubes for 20 s. The pellet was washed with DPBS and 500 μl room-temperature Accumax Cell Dissociation Solution (Innovative Cell Technologies, San Diego, CA) was added to each microcentrifuge tube. The tubes were incubated in a 37 °C water bath. Every 10 min, the cell pellet was moved through the tip of a fire-polished glass pasteur pipette 20 times. The tubes were placed on ice and 500 μl of DPBS was added after a total of 40 min and the tissue appeared to be 80–90% dissociated. The 1 ml sample was then filtered through a 40um mini cell strainer (pluriSelect, El Cajon, CA) into a sterile, round-bottom, 14 mL (17 × 100 mm) Falcon collection tube (Becton Dickinson, Franklin Lakes, NJ). The microcentrifuge tube used for dissociation was rinsed with 0.5 mL 1× DPBS, and was filtered through the same 40um strainer into the collection tube. The cells were pelleted at 1100 rpm for 10 min at room temperature and resuspended in 70 μl of 1 × PBS + 0.04% BSA. Samples with a viability above 70% were used for the study. 10 × Genomics scRNA-seq was performed by the UCLA Technology Center for Genomics and Bioinformatics (TCGB). 10,000 cells were targeted per run with 50,000 reads per cell on the NextSeq 500 High Output, with the read length being 1 × 75. Please see supplemental Methods S1 for detailed bioinformatic analysis.

### Statistical methods

Monoaminergic neuron, sensory neuron count and fluorescence, microglial structural parameter, and acidification comparisons were analyzed using an unpaired Student’s T-test. The inflammatory cytokine expression changes were analyzed using a one-way ANOVA with Sidak’s multiple comparisons analysis. Monoaminergic neuronal counts with microglial knockdown were analyzed using a one-way ANOVA with Sidak’s multiple comparisons analysis.

### Ethics approval

All animal studies were approved by the UCLA Animal Research Committee.

## Results

### DEPe exposure and neurotoxicity

**Figure 1:**
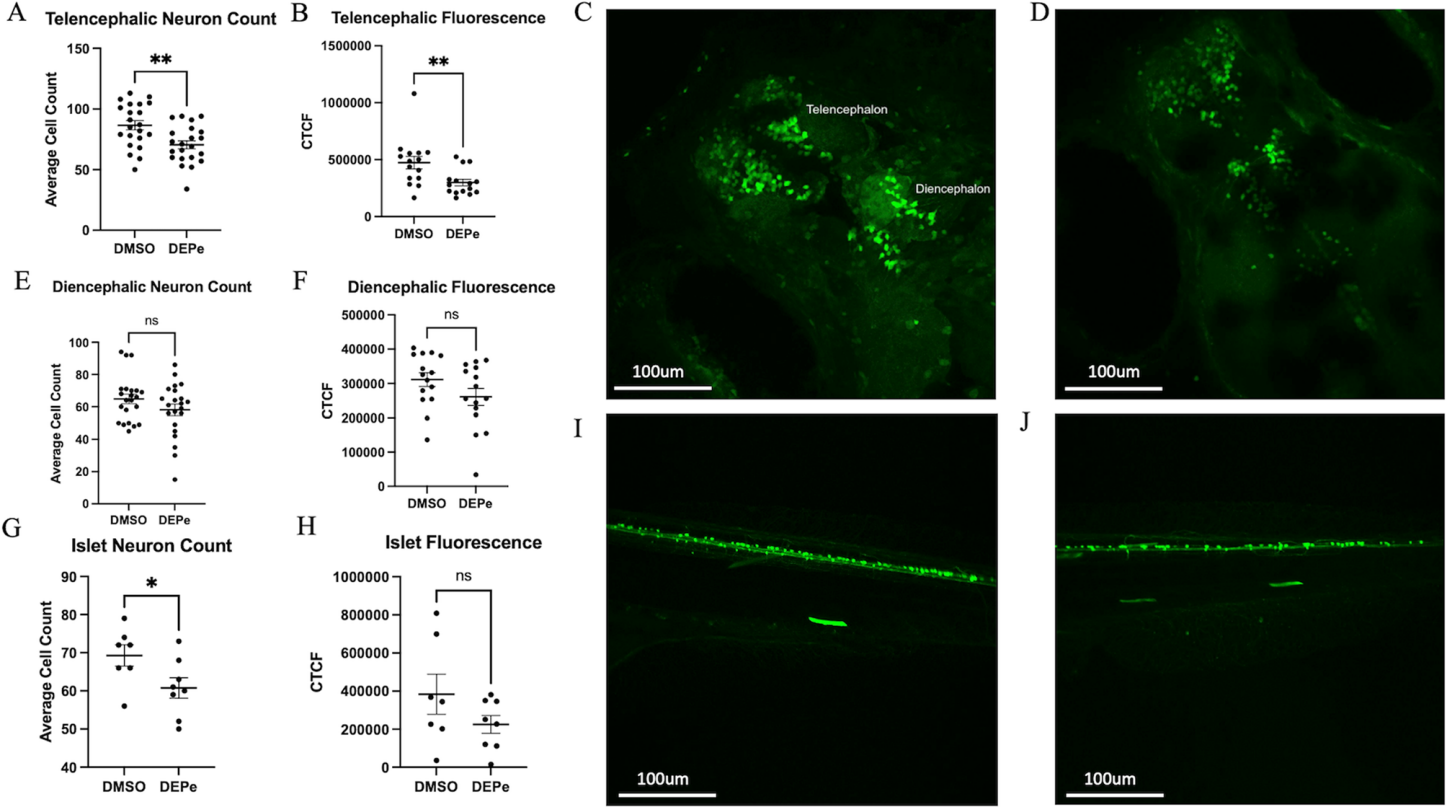
DEPe-induced neurotoxicity. Significant loss of telencephalic neurons and GFP fluorescence with DEPe exposure (**A**,**B**). Dorsal view of 5dpf DMSO- (**C**) and DEPe-treated (**D**) vmat2:GFP ZF brains. No significant change in diencephalic neuron numbers (**E**) or fluorescence (**F**) with DEPe exposure. **p < 0.01, n = 22, 15 (DMSO, DEPe). Significant loss of islet neurons with DEPe exposure (**G**) but no significant loss of islet fluorescence (**H**) with DEPe exposure. Lateral view of 5dpf DMSO-treated (**I**) and DEPe-treated (**J**) Tg(isl1[ss]:Gal4-VP16,UAS:eGFP)^zf154^ tail. *p < 0.05, n = 7, 8 (DMSO, DEPe). Student’s T-test; error bars represent SEM.

To determine if DEPe exposure was neurotoxic, we first tested its toxicity in vivo on monoaminergic neurons using Vmat2:GFP ZF embryos. DEPe exposure resulted in a 20% loss (p = 0.003) of neurons in the telencephalon along with a 37% (p = 0.008) decrease in fluorescence intensity (Fig. 1A–D). The diencephalic neurons exhibited a loss of 10% (p = 0.15) along with a 16% decrease in fluorescence intensity (p = 0.12), which did not reach statistical significance (Fig. 1E,F). In the sensory *isl1* neurons, there was a 12% loss (p = 0.047) by count with DEPe exposure, although the loss in fluorescence was not significant (p = 0.17) (Fig. 1G–J). The non-selective neurotoxicity reported here are similar to those we reported using a different source of DEPe and exposure paradigm^9^.

### Microglial structural and functional activation after DEPe exposure

**Figure 2:**
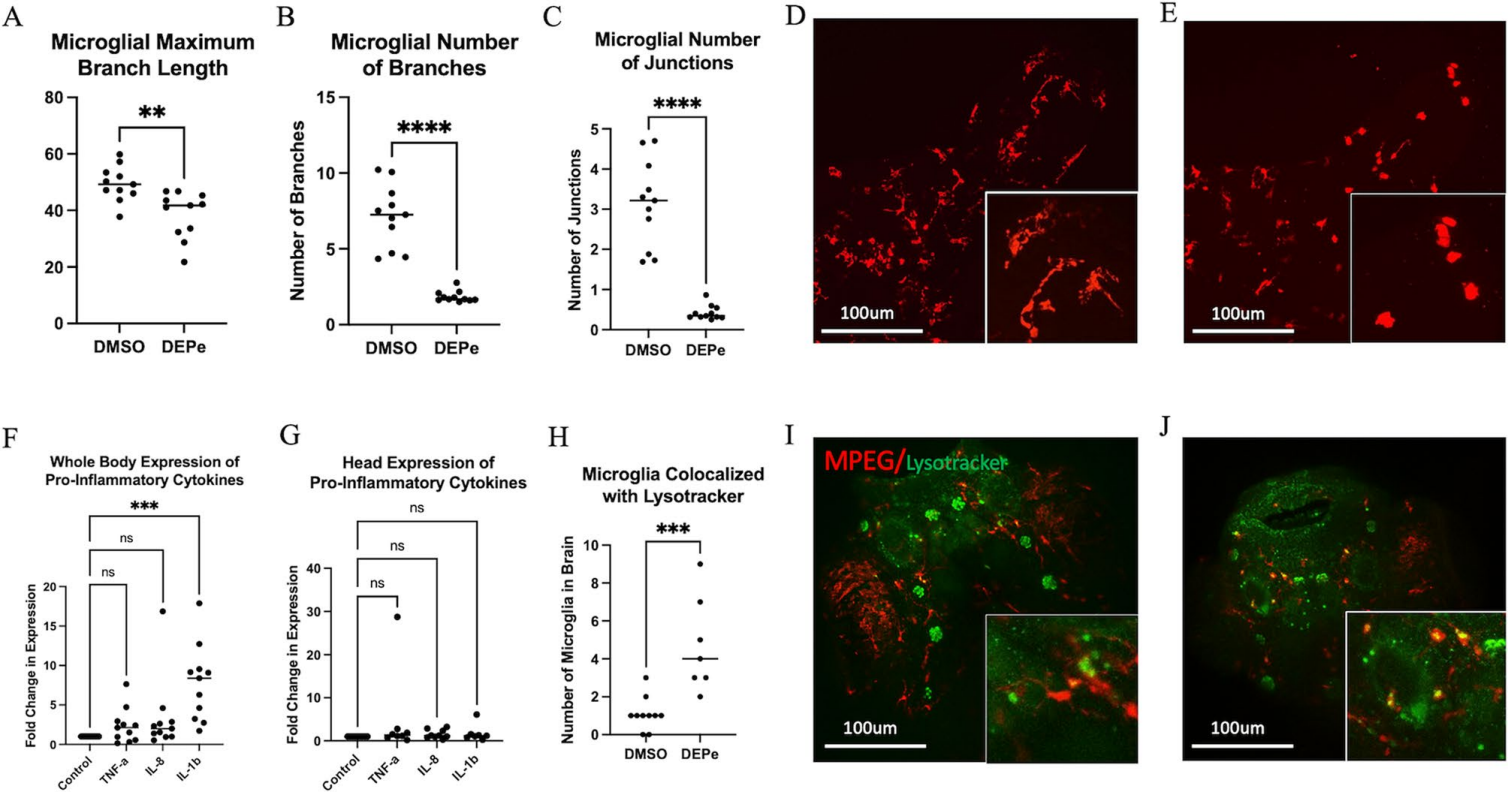
Microglial activation with DEPe exposure. Maximum branch length was significantly shorter (**A**), fewer numbers of branches (**B**) and fewer junctions (**C**) with DEPe exposure. Student’s T-test, **p < 0.01, ****p < 0.0001, n = 11, 11 (DMSO, DEPe). Dorsal view of 5dpf mpeg1:mCherry ZF brains revealing that control microglia (**D**) had more processes and were less rounded than DEPe-treated cells (**E**). Whole-body expression of pro-inflammatory cytokines with DEPe exposure, IL-1b expression was significantly upregulated compared to DMSO (**F**). There were no significant changes in expression of pro-inflammatory cytokines with DEPe exposure in the heads of ZF (**G**). For F and G, one-way ANOVA with Dunnett’s test, ***p < 0.001, n = 5 samples. Significantly more microglial cells colocalized with lysotracker-labeling 5dpf in DEPe-treated microglia (**H**,**J**) compared to DMSO-treated controls (**H**,**I**). Student’s T-test, ***p < 0.001, n = 10, 7.

We characterized microglial response to DEPe through structural and functional analysis. Microglia in DEPe-exposed animals appeared more rounded with shorter processes (Fig. 2A–E). Branch length, number of branches, and number of junctions were all significantly decreased (Fig. 2A–C). These structural changes in microglia are consistent with a more activated state^16^.

Microglia have been observed to acidify lysosomes when activated^17^. We live-imaged the DEPe-induced acidification of microglial lysosomes using lysotracker dye (Fig. 2H–J). We quantified the number of microglia per brain that co-localized with lysotracker labeling and found a significant increase following DEPe exposure, from 1.1 to 4.7 microglia per brain on average (p = 0.0007; Fig. 2H). Thus, DEPe induced structural and functional changes consistent with activated microglia in ZF brains.

### Expression of Pro-inflammatory cytokines after DEPe exposure

To determine the extent of inflammation in the ZF larvae after DEPe treatment, we quantified systemic and head-specific changes in expression of inflammatory cytokines TNF-α, IL-8, and IL-1ß. Systemically, only IL-1ß expression was significantly altered, resulting in an increase after DEPe exposure (Fig. 2F; p = 0.0002). Using RNA collected from the heads, we observed no significant changes in the expression of the three pro-inflammatory cytokines after DEPe treatment (Fig. 2G).

### Neuron injury in the context of microglial knockdown

In order to determine the role microglia might be playing in DEPe-induced neurotoxicity, we blocked microglial development before DEPe exposure. pU1 morpholino injection completely inhibited mpeg1:mcherry-positive microglia in the brain through 72 hpf (Suppl Fig. S1A–C). The loss of telencephalic neurons with DEPe treatment were: 20% loss with no injection, 26% loss with control morpholino (scrambled) injection, and 21% loss with pU1 injection. Morpholino injection by itself was toxic but there was no significant difference between the number of surviving neurons in the control injected and pU1 injected embryos (Suppl Fig. S1D). Additionally, in the diencephalon, there were no significant changes in the number of surviving neurons across any of the conditions (Suppl Fig. S1E). These results suggest that, in this model, microglia do not play a direct role in the subacute aminergic neuronal injury observed with DEPe exposure, but does raise the question of what are the consequences of microglia activation. For this reason, we performed single cell RNA sequencing (scRNAseq) in DEPe-exposed and control animals to better understand if these activated microglial are detrimental or beneficial.

### Single-cell analysis

A total of 80,075 cells were analyzed from 3 biological replicates of DMSO (40,757 cells) and DEPe-treated (39,948 cells) larval heads. Twenty-eight transcriptionally distinct clusters were identified. All clusters were represented by cells from all biological replicates (Suppl Fig. S2A–C). The cell types and markers are listed in Supplementary Table S3.

### Microglial cluster analysis

**Figure 3:**
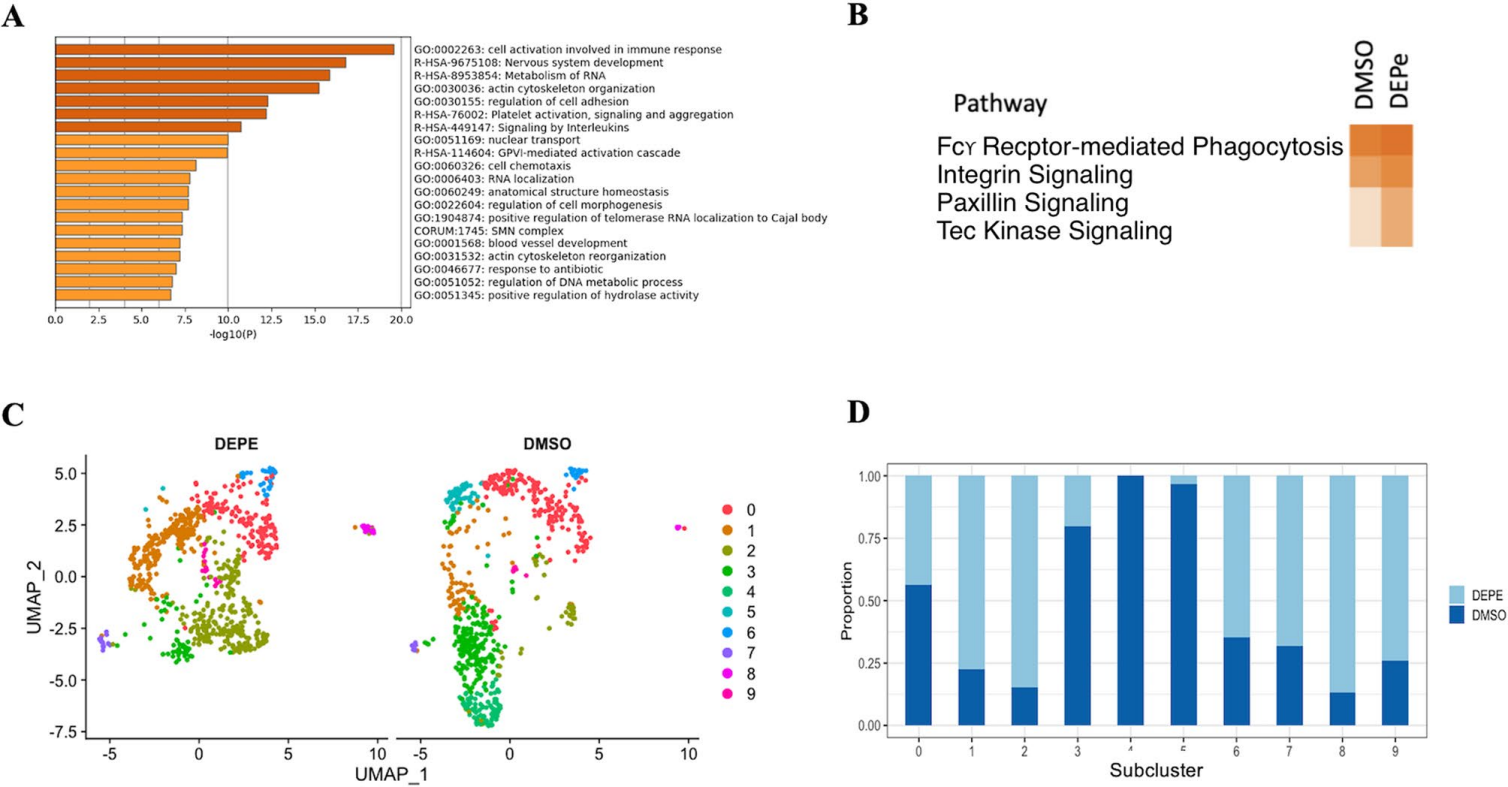
Microglial cluster analysis with and without DEPe exposure. (**A**) The top enriched biological processes for control microglia. (**B**) Ingenuity Pathways Analysis summary. Darker orange denotes more activated pathways (z-score > 2 and p-value < 0.05). (**C**) UMAP plots of DEPe and DMSO treated microglia subclusters. (**D**) Subclustered microglia distribution with DEPe and DMSO treatments.

The microglial cluster (Cluster 11) was identified using the marker genes: ccl34b.1, mfap4, and mpeg1.1 (Suppl Fig. S2C)^18^. The transgenic mCherry fluorophore expression was also assessed, but not relied upon as a marker due to its very low expression compared to the marker genes. The most enriched biological processes identified by GSEA were cell activation involved in immune response, nervous system development, metabolism of RNA, actin cytoskeleton organization, and regulation of cell adhesion (Fig. 3A).

Using IPA Comparison Analysis, we identified microglial pathways that exhibited the greatest changes in activation state with DEPe treatment. They were Integrin Signaling (more active; z-score > 2 and p-value < 0.05), Paxillin Signaling (more active; z-score > 2 and p-value < 0.05), Fcγ Receptor-Mediated Phagocytosis in Macrophages and Monocytes Pathway (more active; z-score > 2 and p-value < 0.05), and Tec Kinase Signaling Pathway (more active; z-score > 2 and p-value < 0.05). Integrin and paxillin signaling influence microglial cytoskeleton structure, migration, and adhesion^19^. In addition, Fcγ receptor binding initiates a signaling cascade, leading to expression of several lysosomal genes, including those involved in acidification^20^. Most Tec Kinases modulate responses to external stimuli^21^ and are associated with Fcγ Receptor-induced phagocytosis^22^. Thus, DEPe exposure resulted in histological evidence of microglial activation which is supported by scRNA-seq analysisas demonstrated by the upregulation of pathways regulating microglial structure and lysosomal function.

We identified 55 significantly altered microglial differentially expressed genes (DEG) with DEPe treatment (Table 1). Grn2 (5.57-fold) exhibited the highest change in expression and is associated with healthy microglia (see Suppl Table S4 for references for alterations in gene expression). Interestingly, mutations in the progranulin gene lead to premature degradation of granulin mRNAs and cause frontotemporal dementia. We also observed the upregulation of g0s2 (1.82-fold), apoeb (1.53-fold), and *ccr9a* (1.30-fold). Their upregulation has been observed in microglia in inflammatory conditions of the nervous system including AD. Interestingly, the anti-inflammatory cytokine il13 (1.78-fold) exhibited increased expression as well and *cd74a* (0.73-fold), a marker of activated microglia, was downregulated. Both of these changes suggest there is a balance between inflammatory and anti-inflammatory responses after exposure to DEPe. Because many disease- and activation-relevant gene expression changes are consistent with a heterogeneous microglial response to DEPe, we performed subcluster analysis to identify microglial sub-states in an attempt to further describe the response to DEPe.

**Table 1:** Microglial DEG with DEPe exposure.

	Fold change		Fold change
grn2	5.574380555	CABZ01058647.1	1.347354042
icn	2.362166957	si:dkey-21e2.16	1.34449382
scinla	1.894250982	srgn	1.335552148
si:ch211-14a17.10	1.893145062	CABZ01021592.1	1.333724761
pvalb5	1.874865643	si:dkey-21e2.14	1.331168596
g0s2	1.815755982	si:dkey-21e2.10	1.329789289
il13	1.781904257	fabp1b.1	1.327708335
thy1	1.637391784	BX322787.1	1.327291235
apoc1	1.608713353	tfa	1.323033492
si:dkey-21e2.3.1	1.578870472	apoa1b	1.321287447
hbbe1.1	1.548410468	mylpfa	1.312756467
si:ch211-207n23.2	1.540890506	actc1b	1.309014809
apoeb	1.527787026	ccl20a.3	1.308253707
il17a/f2	1.509928653	krt5	1.306298023
si:dkey-247k7.2	1.483516228	ccr9a	1.298837197
myhz1.1	1.472410126	elavl4	1.293876822
cst14b.2	1.464081817	atp2a1	1.29314754
si:dkey-21e2.15	1.451568216	si:ch73-359m17.9	1.291737356
si:ch211-212d10.1	1.436802272	si:dkey-21e2.8	1.291196361
hbbe2	1.422749262	nkl.1	0.777343688
FP236331.1	1.419917825	lgals3bpb	0.776339154
s100a11	1.419093518	pgam1a	0.771836684
ifi30	1.40363528	si:ch211-161c3.6	0.76424604
agr2	1.394821988	cxcr4b	0.73150926
si:ch73-359m17.7	1.388832686	cd74a	0.728394705
cpa5	1.375196459	fthl28	0.685357349
pdia2	1.357693902	star	0.400666768
and2	1.347875299		

We identified 10 microglial subclusters (Fig. 3C,D, Table 2). 6 contained enough cells (n > 50) for characterization. Subcluster 0 consisted of a comparable number of DMSO and DEPe treated cells (181 vs. 141) and was characterized as activated due to the expression of various genes related to microglial activation, such as cd74a, ccl34b.1, s100a10b, and il1b. Microglia in this subcluster highly upregulated one of the overall DEG with DEPe, ifi30, which has been observed to be highly upregulated in IFN γ-activated microglia that co-express MHC Class II. This is validated by the high co-expression of *mhc2a* in this Subcluster. Subcluster 1 was more common in DEPe-treated microglia (71 vs. 244) and appears to be disease relevant. For example, *ccl20a.3* expression has been reported to be induced more than threefold after amyloid beta exposure and *ccr9a* has been described *as* a marker of neurotoxic microglia. Subcluster 2 was also more common in DEPe treated microglia (46 vs. 258) and appear to be associated primarily with preventing inflammatory pathology rather than enhancing it based on the relative reduced expression of several genes including ifi30, ccr9a, ccl20a.3, apoeb, lgals2a, and cd63. Interestingly, Subcluster 3 was more common in DMSO-treated microglia than DEPe (196 vs. 50). They expressed genes that parallel patterns observed in activated microglia under experimental conditions involving ischemia and LPS exposure, such as ahcy, myca, rpl3, rplp0, and rps3a*.* Subcluster 4 consisted of only 87 DMSO-treated microglia that appeared to be progenitor-like and hematopoietic, due to the high expression of blf and znfl2a. In addition, DEG with DEPe, srgn, mylpfa, and apoa1b were all significantly downregulated in this subcluster. Subcluster 5 contained many DMSO treated microglia (60 vs. 2), and these cells appeared to be progenitor-like as well, due to the high expression of shha and shhb. Subcluster 6–10 did not have enough cells for accurate interpretation.

**Table 2:** Microglial subcluster analysis.

Subcluster	#cells DMSO	#cells DEPE	Selected marker genes
0	181	141	Up: lygl1, mfap4, ctsl.1, ccl34a.4, mhc2a, ctss2.2, irf8, spi1a, c1qa, ccl34b.1
1	71	244	Up: ccl20a.3, ccl38a.5, ccl36.1, ccr9a, hsp70.3, hsp70.2, ccl34b.1
2	46	258	Up: tmsb4x, rac2, cebpb Down: lgals2a, cebpa, cebpb, cd63
3	196	50	Up: myca, ahcy, rps3a, cirbpb, rpl3, rplp0, tcp1, rack1, hsp90ab1, rpl7, rpl18a, rpl10, rpl11, rpl10a, rpl12
4	87	0	Up: blf, fthl1a, prdx2
5	60	2	Up: shha, shhb, cfd
6	17	31	Up: apoeb, ccl34b.1, lgals2a, g0s2, ctsz, cd63, ctsa
7	8	17	Up: il4, il17, gata2a
8	3	20	Up: grn2, cd9b, cd63
9	6	17	Up: npsn, lyz, mmp13a, mpx

In summary, subclustering revealed sub-states of microglia that appear potentially damaging (Subcluster 0, 1, and 3), protective (Subcluster 2), and progenitor-like (Subcluster 4 and 5). This analysis also demonstrates that DEPe exposure can alter genes relevant to microglial activation states, as demonstrated in the tendency of microglia from treated animals to cluster differentially (Subcluster 1 and 2). Functional assays are needed to fully characterize the activities of these subclusters beyond quantitating gene expression profiles.

### Astroglial cluster analysis

The astroglial cluster (Cluster 9) was identified using the markers: slc1a2b, gfap, s100b (Suppl Fig. S3). The low and mosaic eGFP expression under the gfap promoter using the plasmid injection was not used as a marker. The 5 most enriched biological processes in the astroglia via GSEA were eukaryotic translation elongation, brain development, cell projection morphogenesis, developmental growth, and response to growth factor (Suppl Fig. S3A).

IPA Comparison Analysis identified astroglial pathways that exhibited the greatest changes in activation with DEPe treatment. Relevant pathways were Pregnane X Receptor (PXR) and Aryl Hydrocarbon Receptor (AHR) signaling pathways (less active; z-score < -2 and p-value < 0.05), Paxillin Signaling Pathway (more active; z-score > 2 and p-value < 0.05), and Adrenomedullin Signaling Pathway (more active; z-score > 2 and p-value < 0.05) (Suppl Fig. S3B). The decrease in activation of the PXR and AHR signaling pathways indicate decreased xenobiotic regulation of CYP3A and other Phase I and II metabolic enzyme processes. Activation of the Paxillin Signaling Pathway suggest structural and migratory changes induced by DEPe exposure. Finally, activation of the Adrenomedullin Signaling Pathway indicates the presence of inflammatory cytokines and an activated immune system.

DEPe treatment resulted in 43 significantly altered astroglial DEG (Suppl Table S5). Col10a1a was highly upregulated (1.71-fold), and is a known astrocytic activator^23^. Igfbp1a (1.35-fold) expression in astrocytes has been observed to impair brain development and reduce glial cell proliferation in response to injury. Fabp7b (0.69-fold) has been observed to promote a pro-inflammatory response in astrocytes, harmful for motor neuron survival when upregulated. Hpgd expression was increased 1.39-fold with DEPe unlike the reduced expression previously reported in astrocytes treated with LPS. As with microglia, these changes reflect a complex astroglial response to DEPe exposure and led us to perform subcluster analysis to characterize potentially disease-relevant sub-states.

We identified 12 astroglial subclusters (Suppl Fig. S3C,D, Suppl Table S6). 7 contained enough cells (n > 50) for characterization. Most of the astroglia in Subcluster 0 were in the DEPe treated brains (152 vs. 349). They exhibited significantly downregulated disease-associated genes, such as robo4, ptgdsb.1, ptgdsb.2, and eno1b*.* Subcluster 1 had a comparable number of DMSO and DEPe treated astroglia (206 cells vs. 169 cells), and these microglia, regardless of treatment type, appeared activated. They upregulated many activation-related genes, such as robo4, cxcl14 (increased with TNFa or LPS), cspg5b (upregulated in scar-forming astrocytes), and *mt2* (increased in astrocytes from PD brains). Similarly, astroglia in Subcluster 2 (147 cells vs. 198 cells) appeared to be activated, based on the upregulation of cx43, predicted to be the top key driver of an astrocyte enriched subnetwork associated with AD and which regulates the expression of more than half of known AD risk factor genes mt2, robo4, cspg5a, cspg5b, and cxcl14. Subcluster 3 were more common in DMSO treated brains (211 cells vs. 24 cells) and this astrocyte state appears to be less able to handle stress based on its reduced expression of mt2, robo4, and cxcl14*.* Subcluster 4 contained many more DEPe-treated astroglia (25 cells vs. 205 cells), and these astroglia appeared less activated based on reduced expression of mt2, cxcl14, vim, and ifitm1. Subcluster 5 (45 cells vs. 73 cells) appeared activated and disease associated. The DEPe-treated astroglia in this subcluster clearly upregulated disease-associated genes vim, ifitm1, and col1a2*,* highly expressed in astrocytes in spinal cord injury. Finally, Subcluster 6 appears to be neuroprotective, and was found primarily in DEPe treated brains (12 cells vs. 97 cells). The top marker gene in the DEPe treated cells was expression of prdx1*,* which was highly upregulated in this subcluster similar to that seen in AD and other degenerative diseases and has been proposed to be neuroprotective at least in cell models. Subclusters 7–11 only had 2–29 cells per condition, so they were not interpreted.

In summary, subclustering of astrocytes revealed a complicated response similar to microglia in that some subclusters that became more predominant with DEPe exposure appear to help mitigate some of its toxicity, while other subclusters appear to contribute to it. Whether these subclustering changes impact neuron health after treatment remains to be addressed in a functional manner.

### Neuronal cluster analysis

**Figure 4:**
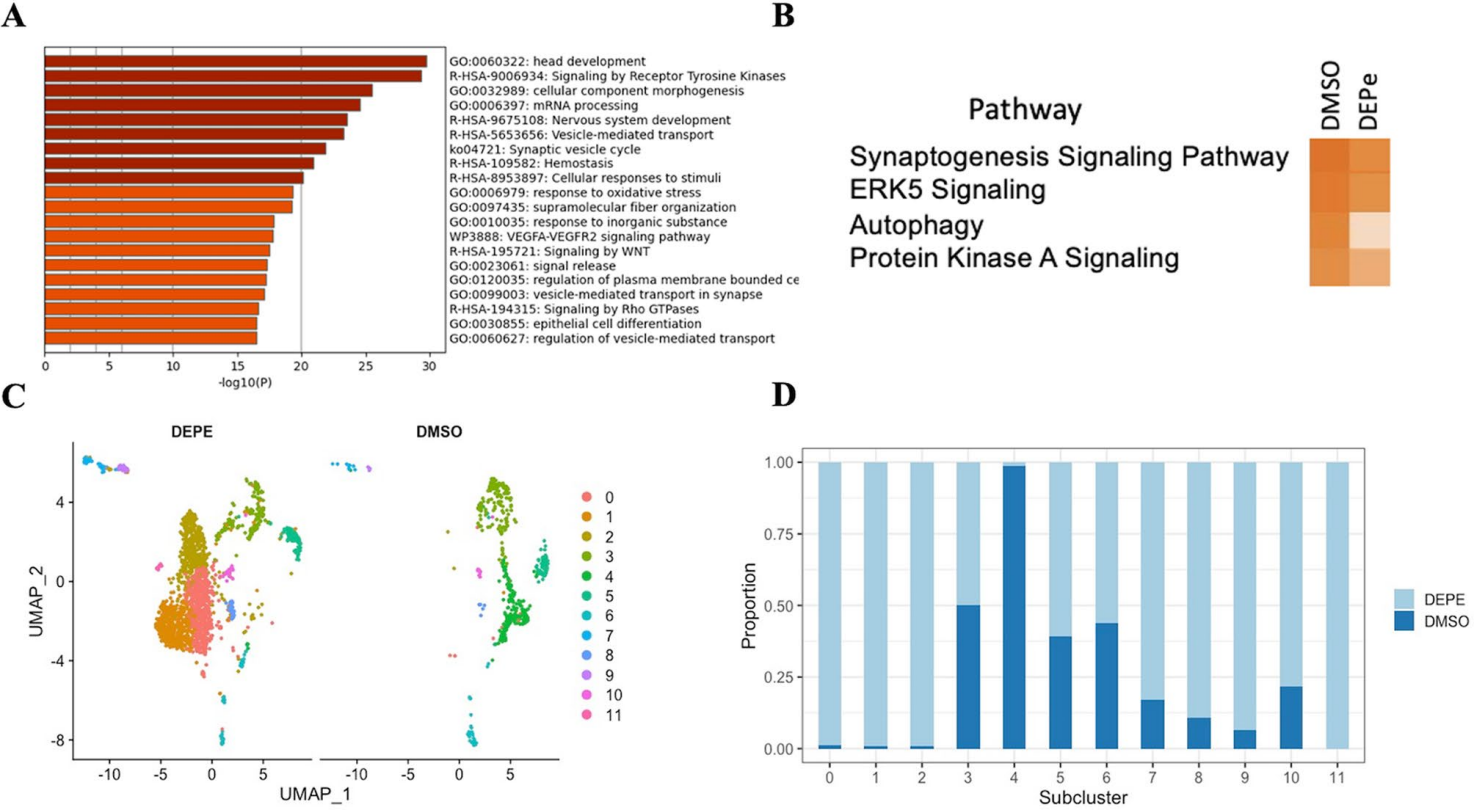
Neuronal cluster 2 analysis with and without DEPe exposure. (**A**) The top enriched biological processes in control neurons. (**B**) Ingenuity Pathways Analysis (IPA) summary. Darker orange denotes more activated pathways (z-score > 2; p-value < 0.05). (**C**) UMAP plots of DEPe and DMSO treated neuronal subclusters. (**D**) Subclustered neuronal distribution with DEPe and DMSO treatments.

Changes in gene expression within neurons specifically was also evaluated after DEPe exposure and 2 neuronal clusters were identified, Cluster 2 and 7 (Fig. 4 and Suppl Figs. S2C and S4). Highly expressed neuronal genes specific to zebrafish neurons included elavl4, orthologous to human neuronal HuD, eno2, orthologous to human neuronal specific enolase, and sncb*,* orthologous to human ß-synuclein. These clusters expressed marker genes that represented several neuron types, such as slc17a6b, orthologous to human vesicular glutamate transporter 2, and gad2, orthologous to human glutamate decarboxylase 2. Of note, the expression of slc18a2 (Vmat2), slc6a2 (dopamine transporter), isl1, and cholinergic neuron markers chata and *ache* were low in both clusters. Filippi et al. reported the co-expression of gad2, slc17a6b and tyrosine hydroxylase in neurons of 4 dpf larvae suggesting that co-expression of markers from different neuronal types is common at this age^24^. Therefore, it was not possible to subcluster neurons by traditional neurotransmitter subtypes. Clusters 2 (neuronal cluster 1) and 7 (neuronal cluster 2) represented the majority of neurons in our study and were further analyzed.

GSEA analysis identified the top 20 enriched biological processes for the two neuronal clusters and both were enriched in processes that emphasized metabolism and responses to external stimuli (Fig. 4A and Suppl Fig. S4A). Neuronal cluster 1 highly expressed many proteolysis and metabolism-related genes, such as prss1, prss59.1, prss59.2, apoda.2, cpa5, nme2b.2, apoa2, apoa1b, cyt1, and apoea. On the other hand, neuronal cluster 2 did not highly express these genes, and instead highly expressed many signaling-related genes, such as mlnl, gngt2b, gng3b, prph, gngt2a, cyt5b, and gng8.

### Neuronal cluster 1 response to DEPe

Pathways within neuronal cluster 1 that exhibited the greatest changes in activation state with DEPe exposure were Oxidative Phosphorylation (less active; z-score < -2 and p-value < 0.05), BEX2 Signaling (less active; z-score < -2 and p-value < 0.05), and Xenobiotic Metabolism PXR and AHR Signaling (more active; z-score > 2 and p-value < 0.05) (Suppl Fig. S4B). Decreased activity of oxidative phosphorylation and BEX2 signaling suggests dysfunction of mitochondria and apoptosis, both have been implicated in the pathogenesis of PD. The increased activation of the Xenobiotic Metabolism Signaling pathways with DEPe exposure is supported by the increased expression of cyp3a65 with DEPe (Suppl Table S7), as AHR and PXR are upstream nuclear receptors of cyp3a65. An interesting disease-related DEG with DEPe treatment was apoeb (1.46-fold), which is orthologous to human APOE, and has been observed to be increased in expression in neurons under pathological conditions and is associated with AD. Overall, DEPe-induced changes in neuronal cluster 1 indicate a decrease in metabolic functions and an increase in activation of xenobiotic metabolism-related pathways.

Neuronal cluster 1 formed 10 subclusters (Suppl Fig. S4C,D, Suppl Table S8). Subcluster 0–6 contained enough cells (n > 50) for interpretation and the majority of neurons in cluster 1 subclustered into subclusters 0, 1 and 2. Some subclustered neurons were more common in DEPe treated brains (e.g. Subcluster 0) while others were found predominantly in control brains (e.g. Subcluster 1). Hindbrain and cerebellar development appear to be inhibited by DEPe given that Subclusters 2 and 5 were low in DEPe-treated brains but beyond this, the significance of the changes in neuronal subclusters was not readily apparent.

### Neuronal cluster 2 response to DEPe

In neuronal cluster 2, DEPe exposure resulted in altered expression of 282 genes related to Synaptogenesis Signaling, ERK5 Signaling, Autophagy, and Protein Kinase A (PKA) Signaling (Fig. 4). The reduction in the autophagy-related genes gabarapa (0.77-fold), map1lc3a (0.81-fold), and map1lc3b (0.77-fold) is particularly noteworthy because we previously found that DEPe exposure reduced autophagic flux in neurons, which was mechanistically linked to DEPe-induced neuronal loss^9^. It has been well established that multiple neurodegenerative diseases exhibit accumulation and ineffective clearance of toxic protein aggregates^25^ and autophagy has been observed to be dysregulated in multiple neurodegenerative diseases including PD^26^, and AD^27^. In addition, there was a significant downregulation of *isl1* (0.79-fold), which aligns with the significant loss of islet neurons quantified in vivo (Fig. 1).

Neuronal Cluster 2 formed 11 subclusters, with Subcluster 0–5 and 7 having enough cells (n > 50) for interpretation (Fig. 4C,D; Suppl Table S9). Subclusters 0, 1, 2 and 5 were over-represented in DEPe-treated brains while subcluster 4 was more prevalent in control brains (Fig. 4D). It is interesting to note that overall expression of synuclein is reduced in neurons with DEPe exposure but is increased in Subcluster 1 (Suppl Tables S7 and S9). In contrast to changes in neuronal cluster 1, subclusters related to cerebellar granule cells were increased (Subcluster 2, Suppl Table S8) with DEPe exposure.

### Olfactory bulb cluster analysis

Cluster analysis revealed the presence of an olfactory bulb cluster (Cluster 19) which is particularly of interest in PD. Olfactory dysfunction and Lewy body pathology in the olfactory bulb are present early in the course of neurodegenerative disorders^28,29^. Furthermore, the olfactory bulb has been shown in vivo to be one of the first areas that ultrafine particle deposition occurs after exposure to AP^30^, and exhibits an inflammatory response with exposure to DE^31^.

Cluster 19 was identified as the olfactory bulb cluster through the identification of marker genes pvalb5*,* which is expressed in the olfactory epithelium, and ompb, orthologous to human olfactory marker protein (OMP). In addition, markers for mitral cells, a subset of olfactory bulb-specific neurons, lhx2a and trpc2b, were also highly expressed in a small proportion of the cluster (Suppl Fig. S2C). The top 5 enriched biological processes via GSEA were response to wounding, embryonic morphogenesis, muscle structure development, VEGFA-VEGFR2 signaling pathway, and response to inorganic substance (Suppl Fig. S5A).

The pathways altered by DEPe in the olfactory bulb cluster included Chemokine Signaling (less active; z-score < -2 and p-value < 0.05) and Unfolded Protein Response (UPR) (more active; z-score > 2 and p-value < 0.05) (Suppl Fig. S5B). Chemokine Signaling is required for neurogenesis in the olfactory epithelium; this has been confirmed in zebrafish and mice^32,33^. With DEPe exposure, neurogenesis may be impaired in the olfactory bulb and contribute to neurodegeneration. Activation of the UPR has been observed in AD and PD through studies of UPR activation markers in human brain tissue^34^. UPR activation maybe a compensatory response to restore protein folding. Lewy bodies contain misfolded α-synuclein and appear early in the olfactory bulb in PD^35^ and increased α-synuclein expression has been described in the olfactory bulb of rodents exposed to DE^7^.

There were 5 significantly altered DEG with DEPe in the olfactory bulb (Suppl Table S10). The most upregulated was cyp1a (3.5-fold), a cytochrome P450 enzyme involved in metabolism of drugs and xenobiotics. cyp1a was the highest induced gene in a transcriptomic and proteomic study of larval zebrafish heads with exposure to DEPe^36^. Our results suggest that the olfactory bulb, which likely has the greatest direct exposure to DEPe in this treatment paradigm, may be primarily responsible for this change. This is an important compensatory mechanism of protection since knocking down cyp1a resulted in an increased sensitivity to DEPe toxicity^36^. Also, there was an upregulation of ugt1b5 (1.36-fold), which is involved in glucuronidation, another pathway involved in the metabolism of toxins and drugs.

## Discussion

The mechanisms by which AP increases the risk of neurodegenerative diseases are unknown but likely include neurotoxicity, neuro- and systemic inflammation, and interactions with the gastrointestinal track^4^. Here, we tested the neurotoxicity and neuroinflammatory response of DEPe, a major component of AP, in a ZF model and found that indeed, it is neurotoxic and induces neuroinflammation. Neuronal loss did not appear selective for dopaminergic neurons and was independent of microglia. Through scRNA-seq analysis, we found that the cellular responses to DEPe were complex and included both compensatory changes in gene expression that would promote survival and detrimental changes that likely contributed to neuronal cell death.

We report here that microglia were activated in response to DEPe based on structural, lysosomal, and gene expression changes. DEG analysis revealed the intricacies of these results, as we observed the upregulation of various disease-associated genes such as g0s2 (G0/G1 Switch Regulatory Protein 2*,* ccr9a (chemokine receptor 9a), and *apoeb (*apolipoprotein Eb), but also anti-inflammatory genes such as il13 (Interleukin 13)*.* These complex responses to DEPe reflect the fact that microglia exist in various states, some of which appear neuroprotective while others likely contribute to neuronal loss^37^. These changes have important implications for chronic exposure, but appear less important in more acute models given the fact that knocking down microglia had no significant effect on neuron loss.

Astroglial responses to toxins are increasingly being recognized as potentially important components to the inflammatory response. Expression analysis also demonstrated that DEPe induced significant shifts in astroglial states related to injury and disease, some likely being protective while others detrimental to neuronal survival. For example, increased expression of gh1 (growth hormone) and col10a1a (collagen, type X, alpha 1a) are involved in regeneration and maybe a compensatory response to counteract neuron loss. Conversely, phase I and II metabolic enzyme processes were downregulated, which would be expected to reduce detoxification of DEPe.

Subacute exposure of DEPe neurotoxicity did not appear to be selective since it resulted in loss of both aminergic and Islet neurons (Fig. 1). This lack of selectivity is consistent with the fact that AP is associated with increased risk of developing PD as well as dementia (including AD). Our scRNA-seq analysis resulted in clusters that possibly reflect 2 states of development (Neuronal clusters 1 and 2), but did not segregate by the transmitters they expressed. For this reason, we were not able to determine if there was selective toxicity based on gene expression. We did find reduced expression of genes involved in oxidative phosphorylation in Neuronal cluster 1 which is of particular interest given that mitochondrial dysfunction has been proposed to be involved in the pathogenesis of PD and AD^38,39^. Xenobiotic metabolism related genes were relatively higher in this cluster compared to controls, possibly reflecting compensatory defenses against the toxins in DEPe. DEPe-induced changes in gene expression in Neuronal cluster 2 were particularly notable since genes controlling synaptogenesis and autophagy were significantly reduced (Fig. 4). We previously reported that DEPe exposure resulted in the accumulation of ɣ1synuclein and reduced neuronal autophagic flux using a live in vivo zebrafish assay^9^. Importantly, reversing this reduction in autophagy with nilotinib protected aminergic neurons. The reduction in expression of autophagy-related genes described here was found using unbiased analysis and adds further support for the hypothesis that dysfunction in proteostasis contributes to DEPe neurotoxicity and maybe a molecular mechanism by which air pollution increases the risk of neurodegeneration.

One of the earliest pathological findings in PD brains is Lewy Body pathology in the olfactory bulb which contain misfolded α-synuclein. Here, DEPe exposure resulted in the upregulation of the unfolded protein response pathway in the Olfactory Bulb Cluster (Suppl Fig. S5). This is of particular interest with respect to neurodegenerative disease since protein misfolding is believed to be critical in the pathogenesis of degenerative disorders. Also of note is that Cyp1a expression was markedly increased in the Olfactory Bulb Cluster in the DEPe-treated fish (Suppl Table S9). We recently reported that Cyp1a expression was increased in whole zebrafish brains with DEPe exposure and knocking down expression resulted in increased DEPe toxicity^36^. The primary route of entry of AP is through inhalation. The olfactory bulb appears to respond to these toxins by upregulating protective pathways that minimize damage.

There are several strengths to this study. The zebrafish model allows for an in vivo evaluation of the effects of subacute exposure to the major components of air pollution which include the evaluation of neuronal toxicity, microglial activation, and the contribution microglia play in the neurotoxicity. Furthermore, we were able to determine the complex transcriptomic inflammatory and neuronal response to DEPe, which identified pathogenic pathways and new potential therapeutic targets.

The zebrafish model also has weaknesses. All studies were performed in developing fish to take advantage of the transparent nature of the larvae, whereas neurodegenerative diseases occur in the aged brain. We feel our findings remain disease-relevant because we are using the adverse outcome pathway approach (AOP) to study disease. It is a well-accepted strategy for studying toxins and disease^40^ and it is particularly important here because it is not possible to study all aspects of a degenerative disease that takes decades to develop in an animal model.

It is important to consider the relevance of DEPe exposures used here to exposure to air pollution. Here, we evaluated the direct toxicity to the brain using relevant concentrations. Lipophilic moieties such as polycyclic aromatic hydrocarbons (PAH) are some of the most toxic components of AP and are known to bioaccumulate in the brain. In a recent study, the concentrations of several PAH were measured in human brains and were very similar to those used in our studies^41^. The mean concentration of 5 PAHs in human brain were between 0.1 to 0.7 ng/g, which were comparable to the levels in our DEPe treatment, 0.3 to 2.2 ng/g (Suppl Table S1). Thus, the concentrations of some of the toxins in DEPe used in this study are at levels that can be seen in the brain chronically.

## Conclusions

DEPe exposure is neurotoxic and induces neuroinflammation. Microglial activation does not contribute to the subacute neurotoxicity, but transcriptomic analysis revealed that microglia and astroglia respond by activating pathways that help protect from further damage as well as pathways that appear to contribute to its toxicity. Furthermore, DEPe reduced expression of genes related to autophagy, consistent with previous studies. These findings add further biological plausibility to the epidemiologic studies connecting air pollution exposure and disease and also have important therapeutic implications. Medications targeting inflammation should be as specific as possible to glia that contribute to cell death, while protecting or enhancing glia that may be neuroprotective. Based on data presented here and a previous study, stimulating neuronal autophagy is another promising therapeutic target.

## Supplementary Information

Supplementary Information 1.

Supplementary Information 2.

Supplementary Information 3.

Supplementary Information 4.

Supplementary Information 5.

Supplementary Information 6.

Supplementary Information 7.

Supplementary Information 8.

Supplementary Information 9.

Supplementary Information 10.

Supplementary Information 11.

Supplementary Information 12.

Supplementary Information 13.

Supplementary Information 14.

Supplementary Information 15.

## Data Availability

All materials and data are available upon request. All requests should be made to Dr. Jeff Bronstein.
