# Supplementary material for: Neurotoxicity of diesel exhaust extracts in zebrafish and its implications for neurodegenerative disease
Source: Sci Rep. 2022 Nov 12;12:19371. doi: 10.1038/s41598-022-23485-2 (PMC9653411; doi:10.1038/s41598-022-23485-2)
Supplement: Supplementary file 1 — Supplementary Information 1. [file 41598_2022_23485_MOESM1_ESM.pdf]

Wisconsin State Laboratory of Hygiene
University of Wisconsin–Madison

Wisconsin State Laboratory of Hygiene
2601 Agriculture Drive, PO Box 7996
Madison, WI 53707-7996
(800)442-4618 - FAX (608)224-6213
http://www.slh.wisc.edu

Laboratory Report

Environmental Health Division

WSLH Sample: 419400001

Report To:	Invoice To:
LORRI TISDALE<br>UCLA<br>10920 WILSHIRE BOULEVARD 5TH F<br>LOS ANGELES, CA 90024-6502	LORRI TISDALE<br>UCLA<br>10920 WILSHIRE BOULEVARD 5TH F<br>LOS ANGELES, CA 90024-6502
	Customer ID: 346313

Field #: JBHM DEPE
Project No:
Collection End: 10/31/2018
Collection Start:
Collected By:
Date Received: 11/13/2018
Date Reported: 12/3/2018
Sample Reason:

ID#:
Sample Location:
Sample Description:
Sample Type:
Waterbody:
Point or Outfall:
Sample Depth:
Program Code:
Region Code:
County:

OC-Air Poll Tracers - Polar

Prep Date: 11/12/18 00:00 Analysis Date: 11/15/18 06:00

Analyte	Analysis Method	Result	Units	LOD	LOQ
Hexanoic Acid	Organic Pollution Tracers	ND	ng/sample	10.0	10.0
Octanoic Acid	Organic Pollution Tracers	ND	ng/sample	10.0	10.0
Decanoic Acid	Organic Pollution Tracers	ND	ng/sample	10.0	10.0
Dodecanoic Acid	Organic Pollution Tracers	43.0	ng/sample	10.0	10.0
Tetradecanoic Acid	Organic Pollution Tracers	160	ng/sample	10.0	10.0
Pentadecanoic Acid	Organic Pollution Tracers	112	ng/sample	10.0	10.0
Hexadecanoic Acid	Organic Pollution Tracers	800	ng/sample	10.0	10.0
Heptadecanoic Acid	Organic Pollution Tracers	139	ng/sample	10.0	10.0
Octadecanoic Acid	Organic Pollution Tracers	1150	ng/sample	10.0	10.0
Nonadecanoic Acid	Organic Pollution Tracers	60.7	ng/sample	10.0	10.0
Pinonic Acid	Organic Pollution Tracers	ND	ng/sample	10.0	10.0
Palmitoleic Acid	Organic Pollution Tracers	ND	ng/sample	20.0	20.0

Wisconsin State Laboratory of Hygiene
UNIVERSITY OF WISCONSIN–MADISON

Wisconsin State Laboratory of Hygiene
2601 Agriculture Drive, PO Box 7996
Madison, WI 53707-7996
(800)442-4618 - FAX (608)224-6213
http://www.slh.wisc.edu

Laboratory Report

Environmental Health Division

WSLH Sample: 419400001

OC-Air Poll Tracers - Polar

Analyte	Analysis Method	Result	Units	LOD	LOQ
Prep Date: 11/12/18 00:00	Analysis Date: 11/15/18 06:00				
Oleic Acid	Organic Pollution Tracers	ND	ng/sample	20.0	20.0
Linoleic Acid	Organic Pollution Tracers	ND	ng/sample	20.0	20.0
Alpha Linolenic Acid	Organic Pollution Tracers	ND	ng/sample	20.0	20.0
Eicosanoic Acid	Organic Pollution Tracers	51.3	ng/sample	10.0	10.0
Heneicosanoic Acid	Organic Pollution Tracers	38.3	ng/sample	10.0	10.0
Docosanoic Acid	Organic Pollution Tracers	71.4	ng/sample	10.0	10.0
Tricosanoic Acid	Organic Pollution Tracers	ND	ng/sample	10.0	10.0
Tetracosanoic Acid	Organic Pollution Tracers	ND	ng/sample	10.0	10.0
Pentacosanoic Acid	Organic Pollution Tracers	ND	ng/sample	10.0	10.0
Hexacosanoic Acid	Organic Pollution Tracers	ND	ng/sample	10.0	10.0
Heptacosanoic Acid	Organic Pollution Tracers	ND	ng/sample	10.0	10.0
Octacosanoic Acid	Organic Pollution Tracers	ND	ng/sample	10.0	10.0
Nonacosanoic Acid	Organic Pollution Tracers	ND	ng/sample	10.0	10.0
Triacontanoic Acid	Organic Pollution Tracers	ND	ng/sample	10.0	10.0
Isopimaric acid	Organic Pollution Tracers	ND	ng/sample	10.0	10.0
Pimaric Acid	Organic Pollution Tracers	ND	ng/sample	10.0	10.0
8,15-Pimaredienoic Acid	Organic Pollution Tracers	ND	ng/sample	1.00	1.00
Dehydroabietic Acid	Organic Pollution Tracers	ND	ng/sample	10.0	10.0
Sandaracopimaric Acid	Organic Pollution Tracers	ND	ng/sample	1.00	1.00
Abietic Acid	Organic Pollution Tracers	ND	ng/sample	10.0	10.0
Abieta-6,8,11,13,15-pentaen-18	Organic Pollution Tracers	ND	ng/sample	10.0	10.0
Abieta-8,11,13,15-tetraen-18-o	Organic Pollution Tracers	ND	ng/sample	10.0	10.0

Wisconsin State Laboratory of Hygiene
UNIVERSITY OF WISCONSIN–MADISON

Wisconsin State Laboratory of Hygiene
2601 Agriculture Drive, PO Box 7996
Madison, WI 53707-7996
(800)442-4618 - FAX (608)224-6213
http://www.slh.wisc.edu

Laboratory Report

Environmental Health Division

WSLH Sample: 419400001

OC-Air Poll Tracers - Polar

Analyte	Analysis Method	Result	Units	LOD	LOQ
Prep Date: 11/12/18 00:00	Analysis Date: 11/15/18 06:00				
7-oxodehydroabietic acid	Organic Pollution Tracers	ND	ng/sample	10.0	10.0
Cholesta-3,5-diene	Organic Pollution Tracers	ND	ng/sample	1.00	1.00
Phthalic Acid	Organic Pollution Tracers	563	ng/sample	10.0	10.0
Isophthalic Acid	Organic Pollution Tracers	ND	ng/sample	10.0	10.0
Terephthalic Acid	Organic Pollution Tracers	ND	ng/sample	10.0	10.0
1,2,4-Benzenetricarboxylic Aci	Organic Pollution Tracers	ND	ng/sample	10.0	10.0
1,2,3-Benzenetricarboxylic Aci	Organic Pollution Tracers	ND	ng/sample	10.0	10.0
1,3,5-Benzenetricarboxylic Aci	Organic Pollution Tracers	ND	ng/sample	10.0	10.0
1,2,4,5-Benzenetetracarboxylic	Organic Pollution Tracers	ND	ng/sample	10.0	10.0
Methylphthalic Acid	Organic Pollution Tracers	ND	ng/sample	10.0	10.0
Malonic Acid	Organic Pollution Tracers	ND	ng/sample	10.0	10.0
Succinic Acid	Organic Pollution Tracers	ND	ng/sample	10.0	10.0
Glutaric Acid	Organic Pollution Tracers	ND	ng/sample	10.0	10.0
Adipic Acid	Organic Pollution Tracers	ND	ng/sample	10.0	10.0
Pimelic Acid	Organic Pollution Tracers	ND	ng/sample	10.0	10.0
Suberic Acid	Organic Pollution Tracers	ND	ng/sample	10.0	10.0
Azelaic Acid	Organic Pollution Tracers	ND	ng/sample	10.0	10.0
Sebacic Acid	Organic Pollution Tracers	ND	ng/sample	10.0	10.0
Maleic Acid	Organic Pollution Tracers	ND	ng/sample	10.0	10.0
Fumaric Acid	Organic Pollution Tracers	ND	ng/sample	10.0	10.0

Wisconsin State Laboratory of Hygiene
UNIVERSITY OF WISCONSIN–MADISON

Wisconsin State Laboratory of Hygiene
2601 Agriculture Drive, PO Box 7996
Madison, WI 53707-7996
(800)442-4618 - FAX (608)224-6213
http://www.slh.wisc.edu

Laboratory Report

Environmental Health Division

WSLH Sample: 419400001

OC-Air Poll Tracers-NonPolar

Prep Date: 11/12/18 00:00 Analysis Date: 11/15/18 06:00

Analyte	Analysis Method	Result	Units	LOD	LOQ
Phenanthrene	Organic Pollution Tracers	220	ng/sample	1.00	1.00
Anthracene	Organic Pollution Tracers	ND	ng/sample	1.00	1.00
Fluoranthene	Organic Pollution Tracers	258	ng/sample	1.00	1.00
Acephenanthrylene	Organic Pollution Tracers	ND	ng/sample	1.00	1.00
Pyrene	Organic Pollution Tracers	ND	ng/sample	1.00	1.00
Methylfluoranthene	Organic Pollution Tracers	ND	ng/sample	1.00	1.00
9-Methylanthracene	Organic Pollution Tracers	ND	ng/sample	1.00	1.00
Benzo(ghi)fluoranthene	Organic Pollution Tracers	142	ng/sample	1.00	1.00
Cyclopenta(cd)pyrene	Organic Pollution Tracers	ND	ng/sample	1.00	1.00
Benz(a)anthracene	Organic Pollution Tracers	73.2	ng/sample	1.00	1.00
Chrysene	Organic Pollution Tracers	56.9	ng/sample	1.00	1.00
1-Methylchrysene	Organic Pollution Tracers	ND	ng/sample	1.00	1.00
Retene	Organic Pollution Tracers	ND	ng/sample	1.00	1.00
Benzo(b)fluoranthene	Organic Pollution Tracers	79.7	ng/sample	1.00	1.00
Benzo(k)fluoranthene	Organic Pollution Tracers	32.2	ng/sample	1.00	1.00
Benzo(j)fluoranthene	Organic Pollution Tracers	ND	ng/sample	1.00	1.00
Benzo(e)pyrene	Organic Pollution Tracers	15.7	ng/sample	1.00	1.00
Benzo(a)pyrene	Organic Pollution Tracers	ND	ng/sample	1.00	1.00
Perylene	Organic Pollution Tracers	ND	ng/sample	1.00	1.00
Indeno(1,2,3-cd)pyrene	Organic Pollution Tracers	ND	ng/sample	1.00	1.00
Benzo(g,h,i)perylene	Organic Pollution Tracers	ND	ng/sample	1.00	1.00
Dibenz(a,h)anthracene	Organic Pollution Tracers	ND	ng/sample	1.00	1.00

Wisconsin State Laboratory of Hygiene
UNIVERSITY OF WISCONSIN–MADISON

Wisconsin State Laboratory of Hygiene
2601 Agriculture Drive, PO Box 7996
Madison, WI 53707-7996
(800)442-4618 - FAX (608)224-6213
http://www.slh.wisc.edu

Laboratory Report

Environmental Health Division

WSLH Sample: 419400001

OC-Air Poll Tracers-NonPolar

Analyte	Analysis Method	Result	Units	LOD	LOQ
Prep Date: 11/12/18 00:00	Analysis Date: 11/15/18 06:00				
Picene	Organic Pollution Tracers	ND	ng/sample	1.00	1.00
Coronene	Organic Pollution Tracers	ND	ng/sample	2.00	2.00
Dibenzo(a,e)pyrene	Organic Pollution Tracers	ND	ng/sample	4.00	4.00
17A(H)-22,29,30-Trisnorhopane	Organic Pollution Tracers	ND	ng/sample	1.00	1.00
17A(H)-21B(H)-30-Norhopane	Organic Pollution Tracers	ND	ng/sample	1.00	1.00
17A(H)-21B(H)-Hopane	Organic Pollution Tracers	ND	ng/sample	1.00	1.00
22S-Homohopane	Organic Pollution Tracers	ND	ng/sample	1.00	1.00
22R-Homohopane	Organic Pollution Tracers	ND	ng/sample	1.00	1.00
22S-Bishomohopane	Organic Pollution Tracers	ND	ng/sample	1.00	1.00
22R-Bishomohopane	Organic Pollution Tracers	ND	ng/sample	1.00	1.00
22S-Trishomohopane	Organic Pollution Tracers	ND	ng/sample	1.00	1.00
22R-Trishomohopane	Organic Pollution Tracers	ND	ng/sample	1.00	1.00
AAA-20S-C27-Cholestane	Organic Pollution Tracers	ND	ng/sample	1.00	1.00
ABB-20R-C27-Cholestane	Organic Pollution Tracers	ND	ng/sample	1.00	1.00
AAA-20R-27-cholestane	Organic Pollution Tracers	ND	ng/sample	1.00	1.00
ABB-20R-C28-Ergostane	Organic Pollution Tracers	ND	ng/sample	1.00	1.00
ABB-20S-C28-Ergostane	Organic Pollution Tracers	ND	ng/sample	1.00	1.00
ABB-20R-C29-Sitostane	Organic Pollution Tracers	ND	ng/sample	1.00	1.00
ABB-20S-C29-Sitostane	Organic Pollution Tracers	ND	ng/sample	1.00	1.00
n-Nonane	Organic Pollution Tracers	ND	ng/sample	20.0	20.0
n-Decane	Organic Pollution Tracers	ND	ng/sample	20.0	20.0
n-Undecane	Organic Pollution Tracers	ND	ng/sample	20.0	20.0

Wisconsin State Laboratory of Hygiene
UNIVERSITY OF WISCONSIN–MADISON

Wisconsin State Laboratory of Hygiene
2601 Agriculture Drive, PO Box 7996
Madison, WI 53707-7996
(800)442-4618 - FAX (608)224-6213
http://www.slh.wisc.edu

Laboratory Report

Environmental Health Division

WSLH Sample: 419400001

OC-Air Poll Tracers-NonPolar

Analyte	Analysis Method	Result	Units	LOD	LOQ
Prep Date: 11/12/18 00:00	Analysis Date: 11/15/18 06:00				
n-Dodecane	Organic Pollution Tracers	ND	ng/sample	20.0	20.0
n-Tridecane	Organic Pollution Tracers	ND	ng/sample	20.0	20.0
n-Tetradecane	Organic Pollution Tracers	ND	ng/sample	20.0	20.0
n-Pentadecane	Organic Pollution Tracers	ND	ng/sample	20.0	20.0
n-Hexadecane	Organic Pollution Tracers	ND	ng/sample	20.0	20.0
Norpristane	Organic Pollution Tracers	ND	ng/sample	20.0	20.0
n-Heptadecane	Organic Pollution Tracers	ND	ng/sample	20.0	20.0
Pristane	Organic Pollution Tracers	ND	ng/sample	20.0	20.0
n-Octadecane	Organic Pollution Tracers	ND	ng/sample	20.0	20.0
Phytane	Organic Pollution Tracers	ND	ng/sample	20.0	20.0
n-Nonadecane	Organic Pollution Tracers	ND	ng/sample	20.0	20.0
n-Eicosane	Organic Pollution Tracers	ND	ng/sample	20.0	20.0
n-Heneicosane	Organic Pollution Tracers	ND	ng/sample	20.0	20.0
n-Docosane	Organic Pollution Tracers	ND	ng/sample	20.0	20.0
n-Tricosane	Organic Pollution Tracers	ND	ng/sample	20.0	20.0
n-Tetracosane	Organic Pollution Tracers	ND	ng/sample	20.0	20.0
n-Pentacosane	Organic Pollution Tracers	ND	ng/sample	20.0	20.0
n-Hexacosane	Organic Pollution Tracers	ND	ng/sample	20.0	20.0
n-Heptacosane	Organic Pollution Tracers	ND	ng/sample	20.0	20.0
n-Octacosane	Organic Pollution Tracers	ND	ng/sample	20.0	20.0
iso-Nonacosane	Organic Pollution Tracers	ND	ng/sample	20.0	20.0
Nonacosane	Organic Pollution Tracers	ND	ng/sample	20.0	20.0

Wisconsin State Laboratory of Hygiene
UNIVERSITY OF WISCONSIN-MADISON

Wisconsin State Laboratory of Hygiene
2601 Agriculture Drive, PO Box 7996
Madison, WI 53707-7996
(800)442-4618 - FAX (608)224-6213
http://www.slh.wisc.edu

Laboratory Report

Environmental Health Division

WSLH Sample: 419400001

OC-Air Poll Tracers-NonPolar

Prep Date: 11/12/18 00:00 Analysis Date: 11/15/18 06:00

Analyte	Analysis Method	Result	Units	LOD	LOQ
Anteiso-triacontane	Organic Pollution Tracers	ND	ng/sample	20.0	20.0
Triacontane	Organic Pollution Tracers	ND	ng/sample	20.0	20.0
iso-Hentriacontane	Organic Pollution Tracers	ND	ng/sample	20.0	20.0
Hentriacontane	Organic Pollution Tracers	ND	ng/sample	20.0	20.0
anteiso-Dotriacontane	Organic Pollution Tracers	ND	ng/sample	20.0	20.0
Dotriacontane	Organic Pollution Tracers	ND	ng/sample	20.0	20.0
iso-Tritriacontane	Organic Pollution Tracers	ND	ng/sample	20.0	20.0
Tritriacontane	Organic Pollution Tracers	ND	ng/sample	20.0	20.0
Tetratriacontane	Organic Pollution Tracers	ND	ng/sample	20.0	20.0
Pentatriacontane	Organic Pollution Tracers	ND	ng/sample	20.0	20.0
Hexatriacontane	Organic Pollution Tracers	ND	ng/sample	20.0	20.0
Heptatriacontane	Organic Pollution Tracers	ND	ng/sample	20.0	20.0
Octatriacontane	Organic Pollution Tracers	ND	ng/sample	20.0	20.0
Nonatriacontane	Organic Pollution Tracers	ND	ng/sample	20.0	20.0
Tetracontane	Organic Pollution Tracers	ND	ng/sample	20.0	20.0
Pentadecylcyclohexane	Organic Pollution Tracers	ND	ng/sample	1.00	1.00
Hexadecylcyclohexane	Organic Pollution Tracers	ND	ng/sample	1.00	1.00
Heptadecylcyclohexane	Organic Pollution Tracers	ND	ng/sample	1.00	1.00
Octadecylcyclohexane	Organic Pollution Tracers	ND	ng/sample	1.00	1.00
Nonadecylcyclohexane	Organic Pollution Tracers	ND	ng/sample	1.00	1.00
Squalane	Organic Pollution Tracers	ND	ng/sample	20.0	20.0

Wisconsin State Laboratory of Hygiene
2601 Agriculture Drive, PO Box 7996
Madison, WI 53707-7996
(800)442-4618 - FAX (608)224-6213
http://www.slh.wisc.edu

Laboratory Report

Environmental Health Division

WSLH Sample: 419400001

WDNR LAB ID:113133790 NELAP LAB ID:2091 EPA LAB ID:WI00007, WI00008 WI DATCP ID:105-415

List of Abbreviations:

LOD = Level of detection
LOQ = Level of quantification (for PFAS the LOQ = MRL)
ND = None detected. Results are less than the LOD
F next to result = Result is between LOD and LOQ
Z next to result = Result is between 0 (zero) and LOD
if LOD=LOQ, Limits were not statistically derived

Test results for NELAP accredited tests are certified to meet the requirements of the NELAC standards. For a list of accredited analytes
see http://www.slh.wisc.edu/about/compliance/nelac-laboratory-accreditation
Results, LOD and LOQ values have been adjusted for analytical dilutions and percent moisture where applicable.
Results relate only to the items tested.
This Laboratory Report shall not be reproduced except in full, without written approval of the laboratory.
The water microbiology unit analyzes samples as received and not all samples are tested for preservation before analysis is performed.

Responsible Party

Inorganic Chemistry: Graham Anderson, Supervisor 608-224-6281
Metals: Graham Anderson, Supervisor 608-224-6281
Organics: Erin Mani, Supervisor 608-224-6269
Environmental Toxicology: Dawn Perkins, Supervisor 608-224-6230
Water Microbiology: Martin Collins, Supervisor 608-224-6239
Radiochemistry: David Webb, Division Director 608-224-6227

Wisconsin State Laboratory of Hygiene
University of Wisconsin–Madison

Wisconsin State Laboratory of Hygiene
2601 Agriculture Drive, PO Box 7996
Madison, WI 53707-7996
(800)442-4618 - FAX (608)224-6213
http://www.slh.wisc.edu

Laboratory Report

Environmental Health Division

WSLH Sample: 419400002

Report To:

LORRI TISDALE
UCLA
10920 WILSHIRE BOULEVARD 5TH F
LOS ANGELES, CA 90024-6502

Invoice To:

LORRI TISDALE
UCLA
10920 WILSHIRE BOULEVARD 5TH F
LOS ANGELES, CA 90024-6502

Customer ID: 346313

Field #: JBHM CSE
Project No:
Collection End: 10/31/2018
Collection Start:
Collected By:
Date Received: 11/13/2018
Date Reported: 12/3/2018
Sample Reason:

ID#:
Sample Location:
Sample Description:
Sample Type:
Waterbody:
Point or Outfall:
Sample Depth:
Program Code:
Region Code:
County:

OC-Air Poll Tracers - Polar

Analyte	Analysis Method	Result	Units	LOD	LOQ
Prep Date: 11/12/18 00:00	Analysis Date: 11/15/18 07:07				
Hexanoic Acid	Organic Pollution Tracers	ND	ng/sample	10.0	10.0
Octanoic Acid	Organic Pollution Tracers	ND	ng/sample	10.0	10.0
Decanoic Acid	Organic Pollution Tracers	ND	ng/sample	10.0	10.0
Dodecanoic Acid	Organic Pollution Tracers	ND	ng/sample	10.0	10.0
Tetradecanoic Acid	Organic Pollution Tracers	ND	ng/sample	10.0	10.0
Pentadecanoic Acid	Organic Pollution Tracers	ND	ng/sample	10.0	10.0
Hexadecanoic Acid	Organic Pollution Tracers	48.9	ng/sample	10.0	10.0
Heptadecanoic Acid	Organic Pollution Tracers	ND	ng/sample	10.0	10.0
Octadecanoic Acid	Organic Pollution Tracers	125	ng/sample	10.0	10.0
Nonadecanoic Acid	Organic Pollution Tracers	ND	ng/sample	10.0	10.0
Pinonic Acid	Organic Pollution Tracers	ND	ng/sample	10.0	10.0
Palmitoleic Acid	Organic Pollution Tracers	ND	ng/sample	20.0	20.0

Wisconsin State Laboratory of Hygiene
UNIVERSITY OF WISCONSIN–MADISON

Wisconsin State Laboratory of Hygiene
2601 Agriculture Drive, PO Box 7996
Madison, WI 53707-7996
(800)442-4618 - FAX (608)224-6213
http://www.slh.wisc.edu

Laboratory Report

Environmental Health Division

WSLH Sample: 419400002

OC-Air Poll Tracers - Polar

Prep Date: 11/12/18 00:00 Analysis Date: 11/15/18 07:07

Analyte	Analysis Method	Result	Units	LOD	LOQ
Oleic Acid	Organic Pollution Tracers	ND	ng/sample	20.0	20.0
Linoleic Acid	Organic Pollution Tracers	ND	ng/sample	20.0	20.0
Alpha Linolenic Acid	Organic Pollution Tracers	ND	ng/sample	20.0	20.0
Eicosanoic Acid	Organic Pollution Tracers	ND	ng/sample	10.0	10.0
Heneicosanoic Acid	Organic Pollution Tracers	ND	ng/sample	10.0	10.0
Docosanoic Acid	Organic Pollution Tracers	ND	ng/sample	10.0	10.0
Tricosanoic Acid	Organic Pollution Tracers	ND	ng/sample	10.0	10.0
Tetracosanoic Acid	Organic Pollution Tracers	ND	ng/sample	10.0	10.0
Pentacosanoic Acid	Organic Pollution Tracers	ND	ng/sample	10.0	10.0
Hexacosanoic Acid	Organic Pollution Tracers	ND	ng/sample	10.0	10.0
Heptacosanoic Acid	Organic Pollution Tracers	ND	ng/sample	10.0	10.0
Octacosanoic Acid	Organic Pollution Tracers	ND	ng/sample	10.0	10.0
Nonacosanoic Acid	Organic Pollution Tracers	ND	ng/sample	10.0	10.0
Triacontanoic Acid	Organic Pollution Tracers	ND	ng/sample	10.0	10.0
Isopimaric acid	Organic Pollution Tracers	ND	ng/sample	10.0	10.0
Pimaric Acid	Organic Pollution Tracers	ND	ng/sample	10.0	10.0
8,15-Pimaredienoic Acid	Organic Pollution Tracers	ND	ng/sample	1.00	1.00
Dehydroabietic Acid	Organic Pollution Tracers	ND	ng/sample	10.0	10.0
Sandaracopimaric Acid	Organic Pollution Tracers	ND	ng/sample	1.00	1.00
Abietic Acid	Organic Pollution Tracers	ND	ng/sample	10.0	10.0
Abieta-6,8,11,13,15-pentaen-18	Organic Pollution Tracers	ND	ng/sample	10.0	10.0
Abieta-8,11,13,15-tetraen-18-o	Organic Pollution Tracers	ND	ng/sample	10.0	10.0

Wisconsin State Laboratory of Hygiene
UNIVERSITY OF WISCONSIN–MADISON

Wisconsin State Laboratory of Hygiene
2601 Agriculture Drive, PO Box 7996
Madison, WI 53707-7996
(800)442-4618 - FAX (608)224-6213
http://www.slh.wisc.edu

Laboratory Report

Environmental Health Division

WSLH Sample: 419400002

OC-Air Poll Tracers - Polar

Analyte	Analysis Method	Result	Units	LOD	LOQ
Prep Date: 11/12/18 00:00	Analysis Date: 11/15/18 07:07				
7-oxodehydroabietic acid	Organic Pollution Tracers	ND	ng/sample	10.0	10.0
Cholesta-3,5-diene	Organic Pollution Tracers	ND	ng/sample	1.00	1.00
Phthalic Acid	Organic Pollution Tracers	ND	ng/sample	10.0	10.0
Isophthalic Acid	Organic Pollution Tracers	ND	ng/sample	10.0	10.0
Terephthalic Acid	Organic Pollution Tracers	ND	ng/sample	10.0	10.0
1,2,4-Benzenetricarboxylic Aci	Organic Pollution Tracers	ND	ng/sample	10.0	10.0
1,2,3-Benzenetricarboxylic Aci	Organic Pollution Tracers	ND	ng/sample	10.0	10.0
1,3,5-Benzenetricarboxylic Aci	Organic Pollution Tracers	ND	ng/sample	10.0	10.0
1,2,4,5-Benzenetetracarboxylic	Organic Pollution Tracers	ND	ng/sample	10.0	10.0
Methylphthalic Acid	Organic Pollution Tracers	ND	ng/sample	10.0	10.0
Malonic Acid	Organic Pollution Tracers	ND	ng/sample	10.0	10.0
Succinic Acid	Organic Pollution Tracers	ND	ng/sample	10.0	10.0
Glutaric Acid	Organic Pollution Tracers	ND	ng/sample	10.0	10.0
Adipic Acid	Organic Pollution Tracers	ND	ng/sample	10.0	10.0
Pimelic Acid	Organic Pollution Tracers	ND	ng/sample	10.0	10.0
Suberic Acid	Organic Pollution Tracers	ND	ng/sample	10.0	10.0
Azelaic Acid	Organic Pollution Tracers	ND	ng/sample	10.0	10.0
Sebacic Acid	Organic Pollution Tracers	ND	ng/sample	10.0	10.0
Maleic Acid	Organic Pollution Tracers	ND	ng/sample	10.0	10.0
Fumaric Acid	Organic Pollution Tracers	ND	ng/sample	10.0	10.0

Wisconsin State Laboratory of Hygiene
UNIVERSITY OF WISCONSIN–MADISON

Wisconsin State Laboratory of Hygiene
2601 Agriculture Drive, PO Box 7996
Madison, WI 53707-7996
(800)442-4618 - FAX (608)224-6213
http://www.slh.wisc.edu

Laboratory Report

Environmental Health Division

WSLH Sample: 419400002

OC-Air Poll Tracers-NonPolar

Analyte	Analysis Method	Result	Units	LOD	LOQ
Prep Date: 11/12/18 00:00	Analysis Date: 11/15/18 07:07				
Phenanthrene	Organic Pollution Tracers	ND	ng/sample	1.00	1.00
Anthracene	Organic Pollution Tracers	ND	ng/sample	1.00	1.00
Fluoranthene	Organic Pollution Tracers	ND	ng/sample	1.00	1.00
Acephenanthrylene	Organic Pollution Tracers	ND	ng/sample	1.00	1.00
Pyrene	Organic Pollution Tracers	ND	ng/sample	1.00	1.00
Methylfluoranthene	Organic Pollution Tracers	ND	ng/sample	1.00	1.00
9-Methylanthracene	Organic Pollution Tracers	ND	ng/sample	1.00	1.00
Benzo(ghi)fluoranthene	Organic Pollution Tracers	ND	ng/sample	1.00	1.00
Cyclopenta(cd)pyrene	Organic Pollution Tracers	ND	ng/sample	1.00	1.00
Benz(a)anthracene	Organic Pollution Tracers	ND	ng/sample	1.00	1.00
Chrysene	Organic Pollution Tracers	ND	ng/sample	1.00	1.00
1-Methylchrysene	Organic Pollution Tracers	ND	ng/sample	1.00	1.00
Retene	Organic Pollution Tracers	ND	ng/sample	1.00	1.00
Benzo(b)fluoranthene	Organic Pollution Tracers	ND	ng/sample	1.00	1.00
Benzo(k)fluoranthene	Organic Pollution Tracers	ND	ng/sample	1.00	1.00
Benzo(j)fluoranthene	Organic Pollution Tracers	ND	ng/sample	1.00	1.00
Benzo(e)pyrene	Organic Pollution Tracers	ND	ng/sample	1.00	1.00
Benzo(a)pyrene	Organic Pollution Tracers	ND	ng/sample	1.00	1.00
Perylene	Organic Pollution Tracers	ND	ng/sample	1.00	1.00
Indeno(1,2,3-cd)pyrene	Organic Pollution Tracers	ND	ng/sample	1.00	1.00
Benzo(g,h,i)perylene	Organic Pollution Tracers	ND	ng/sample	1.00	1.00
Dibenz(a,h)anthracene	Organic Pollution Tracers	ND	ng/sample	1.00	1.00

Wisconsin State Laboratory of Hygiene
UNIVERSITY OF WISCONSIN–MADISON

Wisconsin State Laboratory of Hygiene
2601 Agriculture Drive, PO Box 7996
Madison, WI 53707-7996
(800)442-4618 - FAX (608)224-6213
http://www.slh.wisc.edu

Laboratory Report

Environmental Health Division

WSLH Sample: 419400002

OC-Air Poll Tracers-NonPolar

Prep Date: 11/12/18 00:00 Analysis Date: 11/15/18 07:07

Analyte	Analysis Method	Result	Units	LOD	LOQ
Picene	Organic Pollution Tracers	ND	ng/sample	1.00	1.00
Coronene	Organic Pollution Tracers	ND	ng/sample	2.00	2.00
Dibenzo(a,e)pyrene	Organic Pollution Tracers	ND	ng/sample	4.00	4.00
17A(H)-22,29,30-Trisnorhopane	Organic Pollution Tracers	ND	ng/sample	1.00	1.00
17A(H)-21B(H)-30-Norhopane	Organic Pollution Tracers	ND	ng/sample	1.00	1.00
17A(H)-21B(H)-Hopane	Organic Pollution Tracers	ND	ng/sample	1.00	1.00
22S-Homohopane	Organic Pollution Tracers	ND	ng/sample	1.00	1.00
22R-Homohopane	Organic Pollution Tracers	ND	ng/sample	1.00	1.00
22S-Bishomohopane	Organic Pollution Tracers	ND	ng/sample	1.00	1.00
22R-Bishomohopane	Organic Pollution Tracers	ND	ng/sample	1.00	1.00
22S-Trishomohopane	Organic Pollution Tracers	ND	ng/sample	1.00	1.00
22R-Trishomohopane	Organic Pollution Tracers	ND	ng/sample	1.00	1.00
AAA-20S-C27-Cholestane	Organic Pollution Tracers	ND	ng/sample	1.00	1.00
ABB-20R-C27-Cholestane	Organic Pollution Tracers	ND	ng/sample	1.00	1.00
AAA-20R-27-cholestane	Organic Pollution Tracers	ND	ng/sample	1.00	1.00
ABB-20R-C28-Ergostane	Organic Pollution Tracers	ND	ng/sample	1.00	1.00
ABB-20S-C28-Ergostane	Organic Pollution Tracers	ND	ng/sample	1.00	1.00
ABB-20R-C29-Sitostane	Organic Pollution Tracers	ND	ng/sample	1.00	1.00
ABB-20S-C29-Sitostane	Organic Pollution Tracers	ND	ng/sample	1.00	1.00
n-Nonane	Organic Pollution Tracers	ND	ng/sample	20.0	20.0
n-Decane	Organic Pollution Tracers	ND	ng/sample	20.0	20.0
n-Undecane	Organic Pollution Tracers	ND	ng/sample	20.0	20.0

Wisconsin State Laboratory of Hygiene
UNIVERSITY OF WISCONSIN–MADISON

Wisconsin State Laboratory of Hygiene
2601 Agriculture Drive, PO Box 7996
Madison, WI 53707-7996
(800)442-4618 - FAX (608)224-6213
http://www.slh.wisc.edu

Laboratory Report

Environmental Health Division

WSLH Sample: 419400002

OC-Air Poll Tracers-NonPolar

Analyte	Analysis Method	Result	Units	LOD	LOQ
Prep Date: 11/12/18 00:00	Analysis Date: 11/15/18 07:07				
n-Dodecane	Organic Pollution Tracers	ND	ng/sample	20.0	20.0
n-Tridecane	Organic Pollution Tracers	ND	ng/sample	20.0	20.0
n-Tetradecane	Organic Pollution Tracers	ND	ng/sample	20.0	20.0
n-Pentadecane	Organic Pollution Tracers	ND	ng/sample	20.0	20.0
n-Hexadecane	Organic Pollution Tracers	ND	ng/sample	20.0	20.0
Norpristane	Organic Pollution Tracers	ND	ng/sample	20.0	20.0
n-Heptadecane	Organic Pollution Tracers	ND	ng/sample	20.0	20.0
Pristane	Organic Pollution Tracers	ND	ng/sample	20.0	20.0
n-Octadecane	Organic Pollution Tracers	ND	ng/sample	20.0	20.0
Phytane	Organic Pollution Tracers	ND	ng/sample	20.0	20.0
n-Nonadecane	Organic Pollution Tracers	ND	ng/sample	20.0	20.0
n-Eicosane	Organic Pollution Tracers	ND	ng/sample	20.0	20.0
n-Heneicosane	Organic Pollution Tracers	ND	ng/sample	20.0	20.0
n-Docosane	Organic Pollution Tracers	ND	ng/sample	20.0	20.0
n-Tricosane	Organic Pollution Tracers	ND	ng/sample	20.0	20.0
n-Tetracosane	Organic Pollution Tracers	ND	ng/sample	20.0	20.0
n-Pentacosane	Organic Pollution Tracers	ND	ng/sample	20.0	20.0
n-Hexacosane	Organic Pollution Tracers	ND	ng/sample	20.0	20.0
n-Heptacosane	Organic Pollution Tracers	ND	ng/sample	20.0	20.0
n-Octacosane	Organic Pollution Tracers	ND	ng/sample	20.0	20.0
iso-Nonacosane	Organic Pollution Tracers	ND	ng/sample	20.0	20.0
Nonacosane	Organic Pollution Tracers	ND	ng/sample	20.0	20.0

Wisconsin State Laboratory of Hygiene
UNIVERSITY OF WISCONSIN–MADISON

Wisconsin State Laboratory of Hygiene
2601 Agriculture Drive, PO Box 7996
Madison, WI 53707-7996
(800)442-4618 - FAX (608)224-6213
http://www.slh.wisc.edu

Laboratory Report

Environmental Health Division

WSLH Sample: 419400002

OC-Air Poll Tracers-NonPolar

Prep Date: 11/12/18 00:00 Analysis Date: 11/15/18 07:07

Analyte	Analysis Method	Result	Units	LOD	LOQ
Anteiso-triacontane	Organic Pollution Tracers	ND	ng/sample	20.0	20.0
Triacontane	Organic Pollution Tracers	ND	ng/sample	20.0	20.0
iso-Hentriacontane	Organic Pollution Tracers	ND	ng/sample	20.0	20.0
Hentriacontane	Organic Pollution Tracers	ND	ng/sample	20.0	20.0
anteiso-Dotriacontane	Organic Pollution Tracers	ND	ng/sample	20.0	20.0
Dotriacontane	Organic Pollution Tracers	ND	ng/sample	20.0	20.0
iso-Tritriacontane	Organic Pollution Tracers	ND	ng/sample	20.0	20.0
Tritriacontane	Organic Pollution Tracers	ND	ng/sample	20.0	20.0
Tetratriacontane	Organic Pollution Tracers	ND	ng/sample	20.0	20.0
Pentatriacontane	Organic Pollution Tracers	ND	ng/sample	20.0	20.0
Hexatriacontane	Organic Pollution Tracers	ND	ng/sample	20.0	20.0
Heptatriacontane	Organic Pollution Tracers	ND	ng/sample	20.0	20.0
Octatriacontane	Organic Pollution Tracers	ND	ng/sample	20.0	20.0
Nonatriacontane	Organic Pollution Tracers	ND	ng/sample	20.0	20.0
Tetracontane	Organic Pollution Tracers	ND	ng/sample	20.0	20.0
Pentadecylcyclohexane	Organic Pollution Tracers	ND	ng/sample	1.00	1.00
Hexadecylcyclohexane	Organic Pollution Tracers	ND	ng/sample	1.00	1.00
Heptadecylcyclohexane	Organic Pollution Tracers	ND	ng/sample	1.00	1.00
Octadecylcyclohexane	Organic Pollution Tracers	ND	ng/sample	1.00	1.00
Nonadecylcyclohexane	Organic Pollution Tracers	ND	ng/sample	1.00	1.00
Squalane	Organic Pollution Tracers	ND	ng/sample	20.0	20.0

Wisconsin State Laboratory of Hygiene
UNIVERSITY OF WISCONSIN–MADISON

Wisconsin State Laboratory of Hygiene
2601 Agriculture Drive, PO Box 7996
Madison, WI 53707-7996
(800)442-4618 - FAX (608)224-6213
http://www.slh.wisc.edu

Laboratory Report

Environmental Health Division

WSLH Sample: 419400002

WDNR LAB ID:113133790 NELAP LAB ID:2091 EPA LAB ID:WI00007, WI00008 WI DATCP ID:105-415

List of Abbreviations:

LOD = Level of detection
LOQ = Level of quantification (for PFAS the LOQ = MRL)
ND = None detected. Results are less than the LOD
F next to result = Result is between LOD and LOQ
Z next to result = Result is between 0 (zero) and LOD
if LOD=LOQ, Limits were not statistically derived

Test results for NELAP accredited tests are certified to meet the requirements of the NELAC standards. For a list of accredited analytes
see http://www.slh.wisc.edu/about/compliance/nelac-laboratory-accreditation
Results, LOD and LOQ values have been adjusted for analytical dilutions and percent moisture where applicable.
Results relate only to the items tested.
This Laboratory Report shall not be reproduced except in full, without written approval of the laboratory.
The water microbiology unit analyzes samples as received and not all samples are tested for preservation before analysis is performed.

Responsible Party

Inorganic Chemistry: Graham Anderson, Supervisor 608-224-6281
Metals: Graham Anderson, Supervisor 608-224-6281
Organics: Erin Mani, Supervisor 608-224-6269
Environmental Toxicology: Dawn Perkins, Supervisor 608-224-6230
Water Microbiology: Martin Collins, Supervisor 608-224-6239
Radiochemistry: David Webb, Division Director 608-224-6227
