# Supplementary material for: Neurotoxicity of diesel exhaust extracts in zebrafish and its implications for neurodegenerative disease
Source: Sci Rep. 2022 Nov 12;12:19371. doi: 10.1038/s41598-022-23485-2 (PMC9653411; doi:10.1038/s41598-022-23485-2)
Supplement: Supplementary file 2 — Supplementary Information 2. [file 41598_2022_23485_MOESM2_ESM.docx]

**Supplemental Table 2: Sequences for forward and reverse primers**

| Gene | Forward primer sequence | Reverse primer sequence |
| --- | --- | --- |
| tnf-a | GCG CTT TTC TGA ATC CTA CG | TGC CCA GTC TGT CTC CTT CT |
| il-1B | GCC TGT GTG TTT GGG AAT CT | TGA TAA ACC AAC CGG GAC A |
| il-8 | AGC TTG AGG GTC TGG CTG TAG A | GCG TCG GCT TTC TGT TTC A |
| Elf-1a | CTT CTC AGG CTG ACT GTG C | CCG CTA GCA TTA CCC |
