# Supplementary material for: Neurotoxicity of diesel exhaust extracts in zebrafish and its implications for neurodegenerative disease
Source: Sci Rep. 2022 Nov 12;12:19371. doi: 10.1038/s41598-022-23485-2 (PMC9653411; doi:10.1038/s41598-022-23485-2)
Supplement: Supplementary file 3 — Supplementary Information 3. [file 41598_2022_23485_MOESM3_ESM.docx]

**Supplementary Table 3: Cluster identities and markers used for cell type identification**

| Cluster | Cell Type | Markers | Cell Count |
| --- | --- | --- | --- |
| 0 | Pharyngeal arch | ccl25b, cxcl12a, ms4a17a.9 | 6140 |
| 1 | Cornea | hpdb, ogna, thbs4b, col1a1a | 5323 |
| 2 | Neuronal | elavl4, snap25a, sncb | 5103 |
| 3 | Epidermis | cldni, apoeb, pfn | 3155 |
| 4 | Neural progenitor | her15.2, her4.1, her4.2, her15.1 | 2751 |
| 5 | Skeletal muscle | myhz1.1.1, myhb, tnni2a.1 | 2460 |
| 6 | Keratin | cyt1l, icn2 | 2455 |
| 7 | Neuronal | elav14, snap25a, sncb | 2394 |
| 8 | Oligodendrocyte | rbp2a, s100a10a | 2302 |
| 9 | Astroglia | slc1a2b, gfap, s100b | 2055 |
| 10 | Chondrocyte | col2a1a, matn1, col9a2, col9a1a | 1582 |
| 11 | Microglia | cc134b.1, mfap4, mpeg1.1 | 1455 |
| 12 | Erythrocyte | hbae3, hbbe1.1, hbbe1.3 | 1366 |
| 13 | Yolk syncytial layer | tfa, serpina1l | 1330 |
| 14 | Smooth muscle | acta2, tagln, myl9a | 1321 |
| 15 | Liver | rbp2b, ces2a | 1182 |
| 16 | Placode | agr2, muc5.1, cldnh | 1068 |
| 17 | Epithelium | s100a11, cldnh, atp1b1b | 1037 |
| 18 | Eye (non-retina) | krt1-19d, krt92, agr1 | 1016 |
| 19 | Olfactory bulb | pvalb5, ompb, lhx2a, trpc2b | 703 |
| 20 | Muscle | myl10, myl13, myl4 | 631 |
| 21 | Myeloid rostral blood | cdh5, gpr182, sox7, fabp11a, plvapb | 582 |
| 22 | Pigment | defbl1, apoda.1, gpnmb | 502 |
| 23 | Oligodendrocyte 2 | mbpa, cd59, mbpb | 364 |
| 24 | Yolk Syncytial Layer 2 | pvalb9, ifi30, selenop | 264 |
| 25 | Neutrophil | lyz, npsn, srgn, mpx | 260 |
| 26 | Pancreas | prss1, prss59.1, ctrb1, apoda.2 | 174 |
| 27 | Mature glial cell | epd, ggctb, soul5 | 159 |

Note: Cell count indicates number of cells of that cell type in total sample.
