# Supplementary material for: Neurotoxicity of diesel exhaust extracts in zebrafish and its implications for neurodegenerative disease
Source: Sci Rep. 2022 Nov 12;12:19371. doi: 10.1038/s41598-022-23485-2 (PMC9653411; doi:10.1038/s41598-022-23485-2)
Supplement: Supplementary file 4 — Supplementary Information 4. [file 41598_2022_23485_MOESM4_ESM.docx]

**Supplemental Table 4, References for Alterations in Gene Expression**

| *Grn2* | (Yao et al., 2020: Baker, 2006 #3857) |
| --- | --- |
| *g0s2, apoeb, ccr9a* | (Walker, 2020) |
| *il13* | (Shin et al., 2004) |
| *cd74a* | (Hwang et al., 2017) |
| *ccl34b.1* | (Wu et al., 2020) |
| *s100a10b* | (DePaula-Silva et al., 2019) |
| *il1b, ccl20a.3* | (Walker et al., 2006) |
| *ifi30* | (Satoh et al., 2018) |
| *ccr9a* | (Li et al., 2006) |
| *ifi30, ccr9a,* *ccl20a.3*, *apoeb*, *lgals2a*, *cd63* | (Grubman et al., 2021; Walker, 2020) |
| *ahcy* | (McDonough et al., 2020) |
| *myca* | (Pulido-Salgado et al., 2018) |
| *rpl3, rplp0, rps3a* | (Yang et al., 2018) |
| *blf* | (Pimtong et al., 2014) |
| *znfl2a* | (Hogan et al., 2006) |
| *Shha, shhb* | (Pitter et al., 2014) |
| *Col10a1a* | (Neo and Tang, 2017) |
| *Igfbp1a* | (Ni et al., 1997) |
| *Fabp7b* | (Killoy et al., 2020) |
| *Hpgd* | (Madeddu et al., 2015) |
| *robo4, ptgdsb.1, ptgdsb.2* | (Choi et al., 2019) |
| *eno1b* | (You et al., 2020) |
| *robo4* | (Park et al., 2016) |
| *cxcl14* | (Fang et al., 2012) |
| *cspg5b* | (Anderson et al., 2016) |
| *mt2* | (Michael et al., 2011) |
| *mt2*, *robo4*, *cspg5a*, *cspg5b*, *cxcl14* | (Kajiwara et al., 2018) |
| *mt2, cxcl14*, *vim, ifitm1* | (Liu et al., 2014; Zamanian et al., 2012) |
| *vim, ifitm1*, *col1a2* | (Hara et al., 2017) |
| *prdx1* | (Szeliga, 2020) |
| BEX2 | (Li et al., 2017) |
| *cyp3a65* | (Chang et al., 2013) |
| *apoeb* | (Harris et al., 2004) |
| *lhx2a*, *trpc2b* | (Miyasaka et al., 2009) |

**Supplementary References**

Anderson, M.A., Burda, J.E., Ren, Y., Ao, Y., O'Shea, T.M., Kawaguchi, R., Coppola, G., Khakh, B.S., Deming, T.J., and Sofroniew, M.V. (2016). Astrocyte scar formation aids central nervous system axon regeneration. Nature *532*, 195-200.

Chang, C.T., Chung, H.Y., Su, H.T., Tseng, H.P., Tzou, W.S., and Hu, C.H. (2013). Regulation of zebrafish CYP3A65 transcription by AHR2. Toxicol Appl Pharmacol *270*, 174-184.

Choi, D.J., An, J., Jou, I., Park, S.M., and Joe, E.H. (2019). A Parkinson's disease gene, DJ-1, regulates anti-inflammatory roles of astrocytes through prostaglandin D2 synthase expression. Neurobiol Dis *127*, 482-491.

DePaula-Silva, A.B., Gorbea, C., Doty, D.J., Libbey, J.E., Sanchez, J.M.S., Hanak, T.J., Cazalla, D., and Fujinami, R.S. (2019). Differential transcriptional profiles identify microglial- and macrophage-specific gene markers expressed during virus-induced neuroinflammation. J Neuroinflammation *16*, 152.

Fang, J., Han, D., Hong, J., Tan, Q., and Tian, Y. (2012). The chemokine, macrophage inflammatory protein-2gamma, reduces the expression of glutamate transporter-1 on astrocytes and increases neuronal sensitivity to glutamate excitotoxicity. J Neuroinflammation *9*, 267.

Grubman, A., Choo, X.Y., Chew, G., Ouyang, J.F., Sun, G., Croft, N.P., Rossello, F.J., Simmons, R., Buckberry, S., Landin, D.V.*, et al.* (2021). Transcriptional signature in microglia associated with Abeta plaque phagocytosis. Nat Commun *12*, 3015.

Hara, M., Kobayakawa, K., Ohkawa, Y., Kumamaru, H., Yokota, K., Saito, T., Kijima, K., Yoshizaki, S., Harimaya, K., Nakashima, Y.*, et al.* (2017). Interaction of reactive astrocytes with type I collagen induces astrocytic scar formation through the integrin-N-cadherin pathway after spinal cord injury. Nat Med *23*, 818-828.

Harris, F.M., Tesseur, I., Brecht, W.J., Xu, Q., Mullendorff, K., Chang, S., Wyss-Coray, T., Mahley, R.W., and Huang, Y. (2004). Astroglial regulation of apolipoprotein E expression in neuronal cells. Implications for Alzheimer's disease. J Biol Chem *279*, 3862-3868.

Hogan, B.M., Pase, L., Hall, N.E., and Lieschke, G.J. (2006). Characterisation of duplicate zinc finger like 2 erythroid precursor genes in zebrafish. Dev Genes Evol *216*, 523-529.

Hwang, I.K., Park, J.H., Lee, T.K., Kim, D.W., Yoo, K.Y., Ahn, J.H., Kim, Y.H., Cho, J.H., Kim, Y.M., Won, M.H.*, et al.* (2017). CD74-immunoreactive activated M1 microglia are shown late in the gerbil hippocampal CA1 region following transient cerebral ischemia. Mol Med Rep *15*, 4148-4154.

Kajiwara, Y., Wang, E., Wang, M., Sin, W.C., Brennand, K.J., Schadt, E., Naus, C.C., Buxbaum, J., and Zhang, B. (2018). GJA1 (connexin43) is a key regulator of Alzheimer's disease pathogenesis. Acta Neuropathol Commun *6*, 144.

Killoy, K.M., Harlan, B.A., Pehar, M., and Vargas, M.R. (2020). FABP7 upregulation induces a neurotoxic phenotype in astrocytes. Glia *68*, 2693-2704.

Li, H., Gang, Z., Yuling, H., Luokun, X., Jie, X., Hao, L., Li, W., Chunsong, H., Junyan, L., Mingshen, J.*, et al.* (2006). Different neurotropic pathogens elicit neurotoxic CCR9- or neurosupportive CXCR3-expressing microglia. J Immunol *177*, 3644-3656.

Li, P., Ma, K., Wu, H.Y., Wu, Y.P., and Li, B.X. (2017). Isoflavones Induce BEX2-Dependent Autophagy to Prevent ATR-Induced Neurotoxicity in SH-SY5Y Cells. Cell Physiol Biochem *43*, 1866-1879.

Liu, Z., Li, Y., Cui, Y., Roberts, C., Lu, M., Wilhelmsson, U., Pekny, M., and Chopp, M. (2014). Beneficial effects of gfap/vimentin reactive astrocytes for axonal remodeling and motor behavioral recovery in mice after stroke. Glia *62*, 2022-2033.

Madeddu, S., Woods, T.A., Mukherjee, P., Sturdevant, D., Butchi, N.B., and Peterson, K.E. (2015). Identification of Glial Activation Markers by Comparison of Transcriptome Changes between Astrocytes and Microglia following Innate Immune Stimulation. PLoS One *10*, e0127336.

McDonough, A., Noor, S., Lee, R.V., Dodge, R., 3rd, Strosnider, J.S., Shen, J., Davidson, S., Moller, T., Garden, G.A., and Weinstein, J.R. (2020). Ischemic preconditioning induces cortical microglial proliferation and a transcriptomic program of robust cell cycle activation. Glia *68*, 76-94.

Michael, G.J., Esmailzadeh, S., Moran, L.B., Christian, L., Pearce, R.K., and Graeber, M.B. (2011). Up-regulation of metallothionein gene expression in parkinsonian astrocytes. Neurogenetics *12*, 295-305.

Miyasaka, N., Morimoto, K., Tsubokawa, T., Higashijima, S., Okamoto, H., and Yoshihara, Y. (2009). From the olfactory bulb to higher brain centers: genetic visualization of secondary olfactory pathways in zebrafish. J Neurosci *29*, 4756-4767.

Neo, S.H., and Tang, B.L. (2017). Collagen 1 signaling at the central nervous system injury site and astrogliosis. Neural Regen Res *12*, 1600-1601.

Ni, W., Rajkumar, K., Nagy, J.I., and Murphy, L.J. (1997). Impaired brain development and reduced astrocyte response to injury in transgenic mice expressing IGF binding protein-1. Brain Res *769*, 97-107.

Park, J.H., Pak, H.J., Riew, T.R., Shin, Y.J., and Lee, M.Y. (2016). Increased expression of Slit2 and its receptors Robo1 and Robo4 in reactive astrocytes of the rat hippocampus after transient forebrain ischemia. Brain Res *1634*, 45-56.

Pimtong, W., Datta, M., Ulrich, A.M., and Rhodes, J. (2014). Drl.3 governs primitive hematopoiesis in zebrafish. Sci Rep *4*, 5791.

Pitter, K.L., Tamagno, I., Feng, X., Ghosal, K., Amankulor, N., Holland, E.C., and Hambardzumyan, D. (2014). The SHH/Gli pathway is reactivated in reactive glia and drives proliferation in response to neurodegeneration-induced lesions. Glia *62*, 1595-1607.

Pulido-Salgado, M., Vidal-Taboada, J.M., Barriga, G.G., Sola, C., and Saura, J. (2018). RNA-Seq transcriptomic profiling of primary murine microglia treated with LPS or LPS + IFNgamma. Sci Rep *8*, 16096.

Satoh, J.I., Kino, Y., Yanaizu, M., Ishida, T., and Saito, Y. (2018). Microglia express gamma-interferon-inducible lysosomal thiol reductase in the brains of Alzheimer's disease and Nasu-Hakola disease. Intractable Rare Dis Res *7*, 251-257.

Shin, W.H., Lee, D.Y., Park, K.W., Kim, S.U., Yang, M.S., Joe, E.H., and Jin, B.K. (2004). Microglia expressing interleukin-13 undergo cell death and contribute to neuronal survival in vivo. Glia *46*, 142-152.

Szeliga, M. (2020). Peroxiredoxins in Neurodegenerative Diseases. Antioxidants (Basel) *9*.

Walker, D.G. (2020). Defining activation states of microglia in human brain tissue: an unresolved issue for Alzheimer’s disease. . Neuroimmunol Neuroinflammation *7*, 194-124.

Walker, D.G., Link, J., Lue, L.F., Dalsing-Hernandez, J.E., and Boyes, B.E. (2006). Gene expression changes by amyloid beta peptide-stimulated human postmortem brain microglia identify activation of multiple inflammatory processes. J Leukoc Biol *79*, 596-610.

Wu, S., Nguyen, L.T.M., Pan, H., Hassan, S., Dai, Y., Xu, J., and Wen, Z. (2020). Two phenotypically and functionally distinct microglial populations in adult zebrafish. Sci Adv *6*.

Yang, Y., Boza-Serrano, A., Dunning, C.J.R., Clausen, B.H., Lambertsen, K.L., and Deierborg, T. (2018). Inflammation leads to distinct populations of extracellular vesicles from microglia. J Neuroinflammation *15*, 168.

Yao, Y.N., Wang, M.D., Tang, X.C., Wu, B., and Sun, H.M. (2020). Reduced plasma progranulin levels are associated with the severity of Parkinson's disease. Neurosci Lett *725*, 134873.

You, Y., Borgmann, K., Edara, V.V., Stacy, S., Ghorpade, A., and Ikezu, T. (2020). Activated human astrocyte-derived extracellular vesicles modulate neuronal uptake, differentiation and firing. J Extracell Vesicles *9*, 1706801.

Zamanian, J.L., Xu, L., Foo, L.C., Nouri, N., Zhou, L., Giffard, R.G., and Barres, B.A. (2012). Genomic analysis of reactive astrogliosis. J Neurosci *32*, 6391-6410.
