# Supplementary material for: Neurotoxicity of diesel exhaust extracts in zebrafish and its implications for neurodegenerative disease
Source: Sci Rep. 2022 Nov 12;12:19371. doi: 10.1038/s41598-022-23485-2 (PMC9653411; doi:10.1038/s41598-022-23485-2)
Supplement: Supplementary file 5 — Supplementary Information 5. [file 41598_2022_23485_MOESM5_ESM.docx]

**Supplemental Table 4.  Astroglial DEG with DEPe exposure**

|  | Fold Change |  | Fold Change |
| --- | --- | --- | --- |
| gh1 | 4.50204708 | sncga | 0.735563579 |
| BX908782.2 | 2.599339567 | prss59.2 | 0.73396921 |
| rho | 2.128039429 | prss59.1 | 0.725296821 |
| col10a1a | 1.71809239 | ela3l | 0.719098309 |
| si:ch211-133n4.6 | 1.703578434 | atp1a1b | 0.714574585 |
| apoeb | 1.488017504 | slc6a1b | 0.713426329 |
| si:ch73-335l21.4 | 1.452278351 | prss1 | 0.709208868 |
| and2 | 1.395610983 | si:dkey-183j2.10 | 0.705624043 |
| hpgd | 1.38786806 | slc3a2a | 0.705433127 |
| igfbp1a | 1.348486969 | slc7a10b | 0.696050057 |
| sst1.1 | 1.330654731 | fabp7b | 0.694348419 |
| pnocb | 1.327086317 | urp2 | 0.68897906 |
| foxj1a | 1.324569631 | apoda.2 | 0.687316908 |
| pde6ha | 1.320080606 | prss35 | 0.687249623 |
| icn | 1.304306132 | slc6a11b | 0.677916266 |
| rlbp1a | 1.296437477 | sept8b | 0.673571585 |
| serpina1 | 0.775775116 | ela2l | 0.669694862 |
| cyt1l | 0.77576635 | mbpa | 0.669310568 |
| aldocb | 0.774160703 | cpa5 | 0.658313051 |
| gpd1b | 0.76518863 | ela2 | 0.631246022 |
| zgc:112160 | 0.763038493 | efhd1 | 0.606719199 |
| cd81b | 0.739742372 |  |  |
