# Supplementary material for: Neurotoxicity of diesel exhaust extracts in zebrafish and its implications for neurodegenerative disease
Source: Sci Rep. 2022 Nov 12;12:19371. doi: 10.1038/s41598-022-23485-2 (PMC9653411; doi:10.1038/s41598-022-23485-2)
Supplement: Supplementary file 6 — Supplementary Information 6. [file 41598_2022_23485_MOESM6_ESM.docx]

**Supplemental Table 5: Astroglial Subcluster Analysis**

| Subcluster | DMSO | DEPE | Selected marker genes |
| --- | --- | --- | --- |
| 0 | 152 | 349 | Down: cd81a, ptgdsb.1, ptgdsb.2, eno1b, robo4, mfge8a |
| 1 | 206 | 169 | Up: aqp1a.1, ptgdsb.1, ptgdsb.2, mfge8a, cd81b, robo4, cxcl14, cspg5b, eno1b |
| 2 | 147 | 198 | Up: cx43, glula, mt2, slc1a2b, robo4, cspg5b, eno1b, aqp1a.1, cxcl14, cd81a, cspg5a, srgn |
| 3 | 211 | 24 | Down: fkbp5, srgn, cxcl14, mt2, robo4, cx43 |
| 4 | 25 | 205 | Down: mt2, cxcl14, gfap, vim, ifitm1 |
| 5 | 45 | 73 | Up: ifitm1, col1a2, ms4a17a.11, ms4a17a.9, ms4a17a.7, pmp22a |
| 6 | 12 | 97 | Up: prdx1 Down: ptgdsb.1, apoda.2, and eno1b |
| 7 | 17 | 29 |  |
| 8 | 19 | 17 |  |
| 9 | 10 | 19 |  |
| 10 | 2 | 14 | - |
| 11 | 8 | 17 |  |
