# Supplementary material for: Neurotoxicity of diesel exhaust extracts in zebrafish and its implications for neurodegenerative disease
Source: Sci Rep. 2022 Nov 12;12:19371. doi: 10.1038/s41598-022-23485-2 (PMC9653411; doi:10.1038/s41598-022-23485-2)
Supplement: Supplementary file 7 — Supplementary Information 7. [file 41598_2022_23485_MOESM7_ESM.docx]

**Supplementary Table 6: Neuronal DEG with DEPe exposure**

|  | Fold Change |  | Fold Change |
| --- | --- | --- | --- |
| rho | 2.634467006 | apoa1b | 0.824619839 |
| pde6ha | 2.196633961 | rpl24 | 0.823423672 |
| prss59.2 | 2.035960681 | rpl23a | 0.817640301 |
| ptgdsb.1 | 1.882754281 | C20H6orf58.1 | 0.814714717 |
| ela2 | 1.880091115 | rps27.2 | 0.812356409 |
| ela2l | 1.852402206 | hmgb3a | 0.811056892 |
| ctrb1 | 1.795956408 | rps14 | 0.810016119 |
| cela1.6 | 1.792991244 | rps16 | 0.806031836 |
| agr2 | 1.770519511 | ppdpfb | 0.799116048 |
| prss1 | 1.762937499 | rpl34 | 0.79818297 |
| apoa1a | 1.759841754 | gc | 0.79776639 |
| prss59.1 | 1.757284658 | uqcrq | 0.796507343 |
| ela3l | 1.737741377 | uox | 0.796350243 |
| apoda.2 | 1.735779954 | rpl23 | 0.795915067 |
| zgc:111983 | 1.687497731 | rps13 | 0.794860177 |
| si:dkey-247k7.2 | 1.663605526 | hp.1 | 0.792008807 |
| ctrl | 1.649686886 | rpl38 | 0.791796917 |
| c6ast4 | 1.64536156 | aldob | 0.791467933 |
| zgc:136461 | 1.643779808 | rpl27 | 0.790651254 |
| ptgdsb.2 | 1.62901501 | uba52 | 0.788639917 |
| amy2a | 1.627862792 | rps12 | 0.784787965 |
| cel.1 | 1.621638506 | rpl28 | 0.783873908 |
| zgc:112160 | 1.559041735 | cox8a | 0.782969849 |
| hbbe1.3 | 1.523441663 | rps17 | 0.781831765 |
| lgals1l1 | 1.521591834 | cox7b | 0.781379744 |
| cpa5 | 1.518368855 | fetub | 0.777144883 |
| apoa4b.2.1 | 1.507707546 | rpl29 | 0.776468325 |
| si:ch211-207n23.2 | 1.504344929 | rps15 | 0.773902255 |
| pck1 | 1.492677371 | lgals2b | 0.773675036 |
| apoeb | 1.457925064 | rpl22 | 0.772617434 |
| hbae3 | 1.431594924 | myhz1.1 | 0.772071423 |
| sult6b1 | 1.428118851 | rpl32 | 0.771091356 |
| cpb1 | 1.422952694 | rpl30 | 0.769589961 |
| si:dkey-183i3.5 | 1.415808009 | rps18 | 0.764435762 |
| krt5 | 1.411062852 | cyt1l | 0.764298329 |
| apoa4b.1 | 1.403773252 | sec61g | 0.760652318 |
| caspb | 1.399408765 | rps25 | 0.759613578 |
| pdia2 | 1.394531793 | RPS17 | 0.758542236 |
| hbbe1.1 | 1.393267143 | rps19 | 0.75473844 |
| zgc:136930 | 1.352171792 | rplp2 | 0.753372033 |
| agxtb | 1.348705684 | faua | 0.75255188 |
| si:ch211-195b11.3 | 1.335746551 | rpl14 | 0.74863241 |
| her4.1 | 1.335314686 | myl10 | 0.745990633 |
| cfl1l | 1.331973285 | icn | 0.741748669 |
| si:ch211-240l19.5 | 1.32844551 | rplp2l | 0.735883322 |
| zgc:158463 | 1.325277509 | rps29 | 0.734539818 |
| chia.2 | 1.324221644 | rbp2b | 0.732958029 |
| cyp3a65 | 1.320692986 | atp5l | 0.732128326 |
| sycn.2 | 1.319429605 | rps28 | 0.731407603 |
| hmgb2a | 1.30373395 | rpl35 | 0.731363755 |
| cpa4 | 1.301226394 | rps15a | 0.729451004 |
| tnni2b.2 | 1.294685724 | zgc:153409 | 0.726337516 |
| sparc | 1.294280033 | rpl36a | 0.724012794 |
| alas1 | 1.291387414 | si:dkey-151g10.6 | 0.717950914 |
| gsto2 | 1.290415486 | rpl36 | 0.713789733 |
| krt91 | 1.286381565 | fabp10a | 0.70703587 |
| ucp2 | 1.280552191 | rps26l | 0.705511305 |
| hbbe2 | 1.256906072 | rpl31 | 0.704401902 |
| pfn1 | 1.234501866 | rpl37.1 | 0.699605919 |
| krtt1c19e | 1.232659538 | apoc1 | 0.696168482 |
| zgc:92137 | 1.218879381 | sncb | 0.687442284 |
| eef1da | 1.212426853 | zgc:171772 | 0.687367318 |
| tfa | 1.210690149 | hsp90aa1.2 | 0.685146614 |
| fabp7a | 1.205974481 | rps26 | 0.67873245 |
| fth1a | 1.205172503 | rpl39 | 0.677746239 |
| rps27.1 | 0.834610206 | rpl35a | 0.653169241 |
| myl13 | 0.833605235 | pklr | 0.649029612 |
| rplp1 | 0.82765787 | rps21 | 0.633277624 |
| rps23 | 0.827164875 | tcnba | 0.528615308 |
| atp5f1e | 0.824681379 |  |  |
