# Supplementary material for: Neurotoxicity of diesel exhaust extracts in zebrafish and its implications for neurodegenerative disease
Source: Sci Rep. 2022 Nov 12;12:19371. doi: 10.1038/s41598-022-23485-2 (PMC9653411; doi:10.1038/s41598-022-23485-2)
Supplement: Supplementary file 8 — Supplementary Information 8. [file 41598_2022_23485_MOESM8_ESM.docx]

**Supplementary Table 7: Neuronal Cluster 1 Subcluster Data**

| Subcluster | DMSO | DEPE | Selected marker genes | Characterization |
| --- | --- | --- | --- | --- |
| 0 | 164 | 1761 | Slc32a1, gad2, pbx1a, snap25a, gpm6ab, ywhag2, elavl3 | Gabaergic and glutamatergic neurons |
| 1 | 1247 | 78 | Slc6a1b, gad2, snap25a, atp6v0cb | Gabaergic and glutamatergic neurons |
| 2 | 645 | 226 | Neurod1, zic1, zic2a, zic4, zic5 | Granule cell development |
| 3 | 93 | 117 | Hbae3, hbbe1.3, mbpa, myl13, epd, chia.2, krt4 | Unclear |
| 4 | 106 | 120 | Scn4ab, krt18b, tmem176, rgcc, krt8, krt94 | Unclear |
| 5 | 135 | 82 | Vipb, vip, phox2a, tppp2, tlx2, calb2a | Hindbrain, cranial ganglion, Rohon-Beard |
| 6 | 84 | 14 | Ctrl, prss1, ela2, ela2l, ela3, prss59.1 | Unclear |
| 7 | 44 | 13 | - | - |
| 8 | 1 | 45 | - | - |
| 9 | 14 | 28 | - | - |
| 10 | 17 | 9 | - | - |
