# Supplementary material for: Neurotoxicity of diesel exhaust extracts in zebrafish and its implications for neurodegenerative disease
Source: Sci Rep. 2022 Nov 12;12:19371. doi: 10.1038/s41598-022-23485-2 (PMC9653411; doi:10.1038/s41598-022-23485-2)
Supplement: Supplementary file 9 — Supplementary Information 9. [file 41598_2022_23485_MOESM9_ESM.docx]

**Supplementary Table 8: Neuronal Cluster 2 Subcluster Data**

| Subcluster | DMSO | DEPE | Selected marker genes | Characterization |
| --- | --- | --- | --- | --- |
| 0 | 7 | 544 | Krt91, krtt1c19e, pfn1, krt5, ptgdsb.1 | Progenitors, increased structural proteins |
| 1 | 4 | 439 | Slc6a1b, slc32a1, sncgb, atp6v0cb, sncb, snap25a | Gabaergic and glutamatergic neurons, high synuclein |
| 2 | 3 | 408 | Hmgb2a, tcf7l2, zbtb18, sox11a | Progenitor-like neurons, granule cells |
| 3 | 146 | 146 | Isl2a, prph, tppp2, phox2bb, phox2a, tlx2, isl1 | Hindbrain, cranial ganglion, Rohon-Beard |
| 4 | 237 | 3 | Sox4a.1, pou3f1, gpm6aa, lhx2a | Developing gabaergic and glutamatergic neurons |
| 5 | 65 | 101 | Scn4ab, krt18b, rgcc, krt94, vim, krt8, sparc | Epithelial and eye development |
| 6 | 35 | 45 | - | - |
| 7 | 11 | 53 | Rho, opn1mw1, saga, sagb, gnat1, rom1a, rom1b | Retina |
| 8 | 5 | 41 | - | - |
| 9 | 3 | 43 | - | - |
| 10 | 8 | 29 | - | - |
| 11 | 0 | 18 | - | - |
