# Supplementary material for: Neurotoxicity of diesel exhaust extracts in zebrafish and its implications for neurodegenerative disease
Source: Sci Rep. 2022 Nov 12;12:19371. doi: 10.1038/s41598-022-23485-2 (PMC9653411; doi:10.1038/s41598-022-23485-2)
Supplement: Supplementary file 10 — Supplementary Information 10. [file 41598_2022_23485_MOESM10_ESM.docx]

**Supplementary Table 9: Olfactory bulb DEG with DEPe exposure**

|  | Fold Change |
| --- | --- |
| cyp1a | 3.503660121 |
| histh1l | 1.753258062 |
| ptgdsb.1 | 1.57770117 |
| ugt1b5 | 1.357982863 |
| aldob | 0.64861636 |
