# Supplementary material for: Neurotoxicity of diesel exhaust extracts in zebrafish and its implications for neurodegenerative disease
Source: Sci Rep. 2022 Nov 12;12:19371. doi: 10.1038/s41598-022-23485-2 (PMC9653411; doi:10.1038/s41598-022-23485-2)
Supplement: Supplementary file 11 — Supplementary Information 11. [file 41598_2022_23485_MOESM11_ESM.docx]

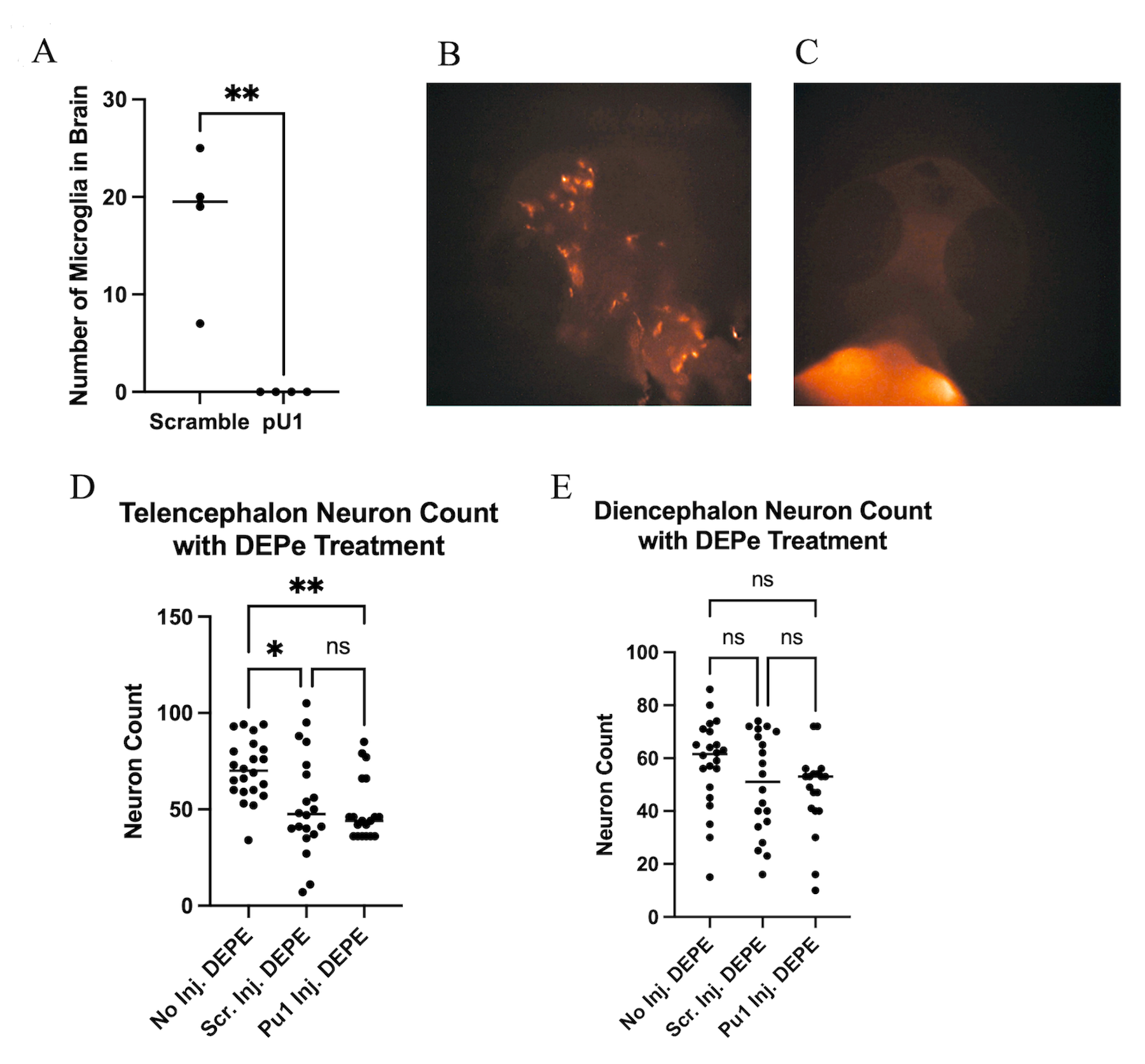


**Supplementary Figure 1**: **Microglia did not contribute to DEPe-induced aminergic neuron loss**. Injection of *pU1* morpholino completely blocked microglial development (A-C). Dorsal view of live 2dpf *mpeg1:mCherry* embryo injected with scramble MO (B) and *pU1* morpholino (C). No significant difference in DEPe-induced aminergic neuron loss in *pU1* and scrambled morpholino injected fish in the telencephalon (D) and diencephalon (E). One-way ANOVA with Sidak’s multiple comparisons test, * = p<0.05, ** = p<0.01, n = 20-22 embryos per condition. All error bars represent SEM.
