# Supplementary material for: Neurotoxicity of diesel exhaust extracts in zebrafish and its implications for neurodegenerative disease
Source: Sci Rep. 2022 Nov 12;12:19371. doi: 10.1038/s41598-022-23485-2 (PMC9653411; doi:10.1038/s41598-022-23485-2)
Supplement: Supplementary file 12 — Supplementary Information 12. [file 41598_2022_23485_MOESM12_ESM.docx]

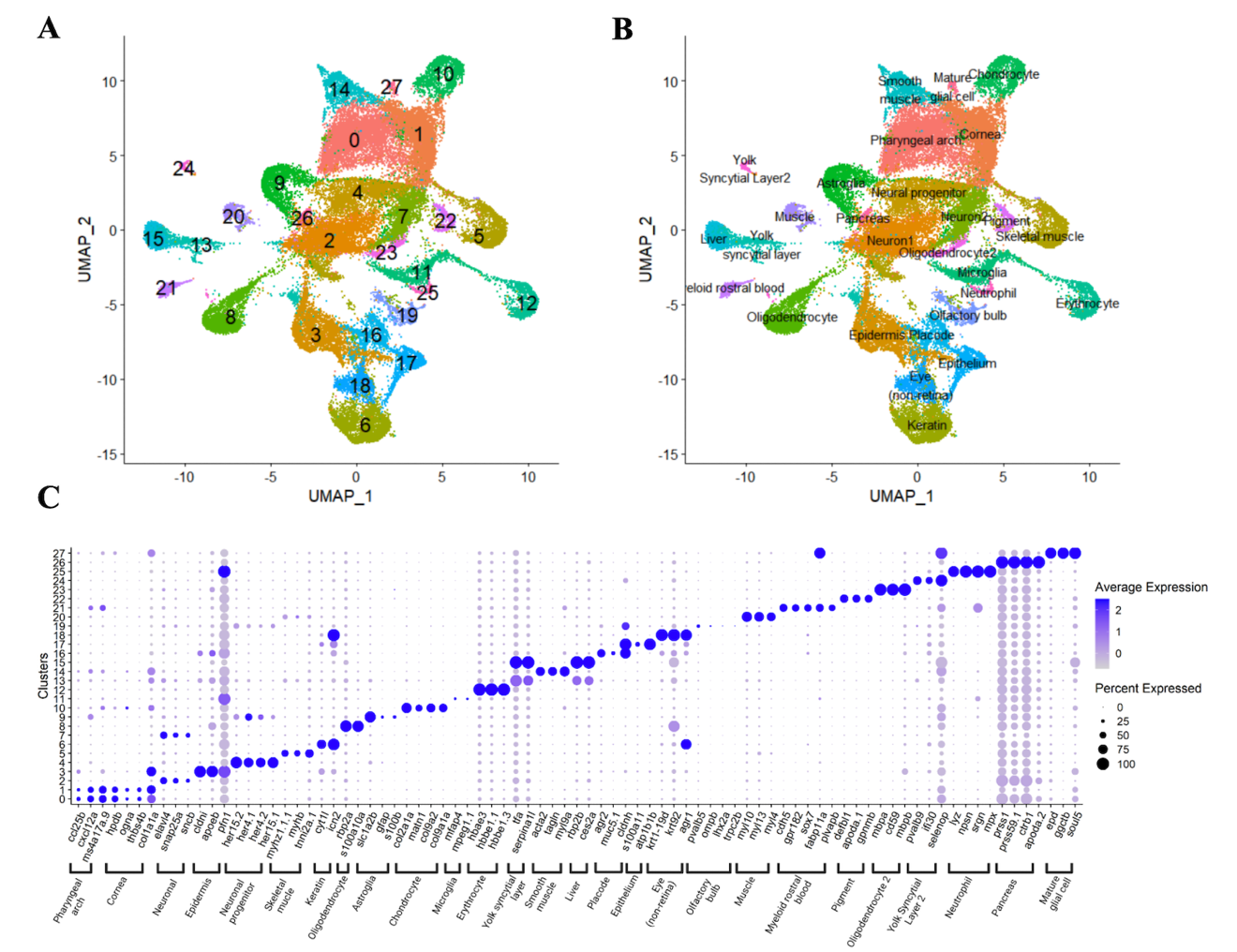


**Supplementary Figure 2: scRNA-seq clustering analysis**. UMAP plot showing the result of (A) clustering analysis and (B) different cell types. C: Dot plot showing the expression of cell-type specific marker genes throughout clusters.
