# Supplementary material for: Neurotoxicity of diesel exhaust extracts in zebrafish and its implications for neurodegenerative disease
Source: Sci Rep. 2022 Nov 12;12:19371. doi: 10.1038/s41598-022-23485-2 (PMC9653411; doi:10.1038/s41598-022-23485-2)
Supplement: Supplementary file 13 — Supplementary Information 13. [file 41598_2022_23485_MOESM13_ESM.docx]

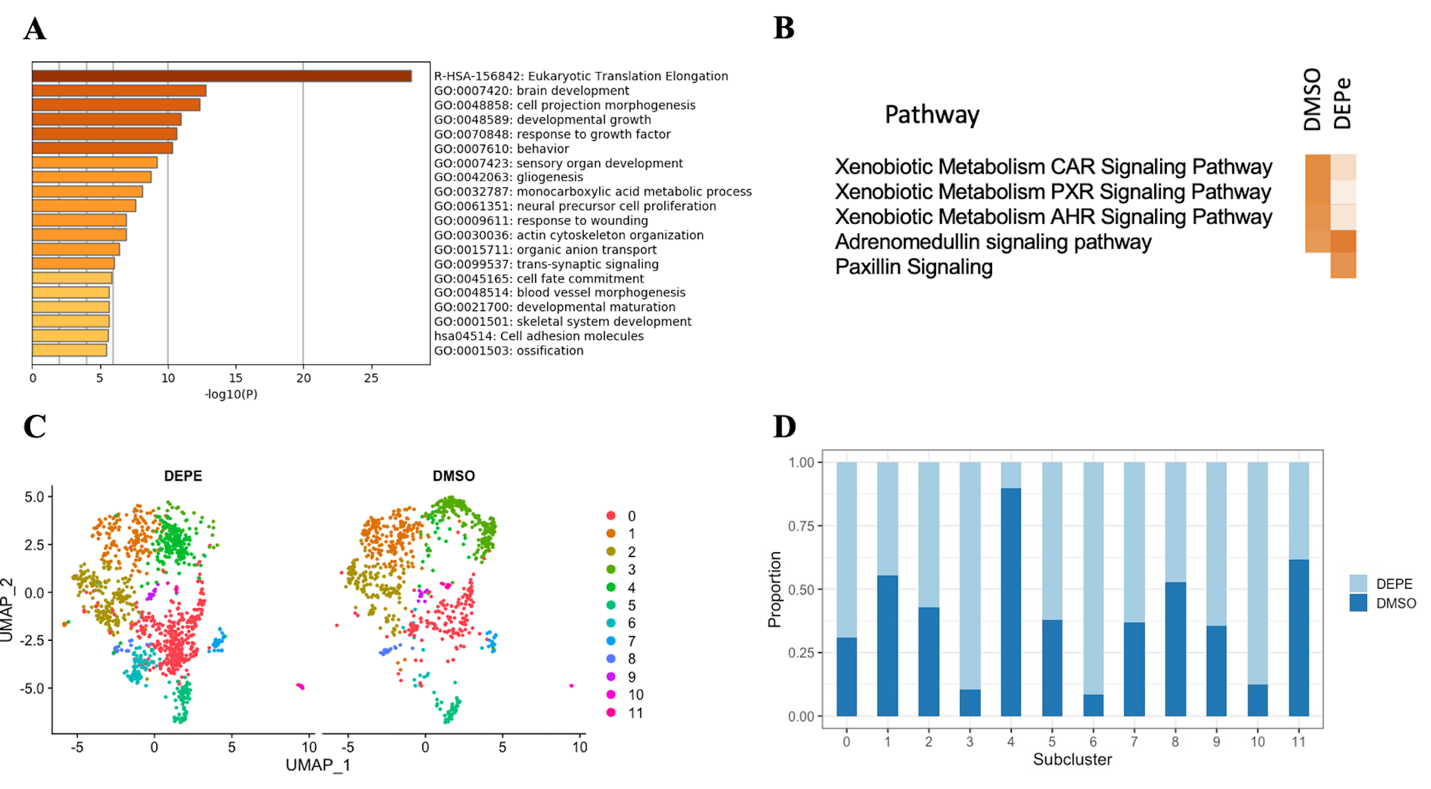


**Supplementary Figure 3:** **Astroglial cluster analysis with and without DEPe exposure**. A: The top enriched biological processes for control astroglia. B: Ingenuity Pathways Analysis summary. Darker orange denotes more activated pathways (z-score > 2; p-value < 0.05). C: UMAP plots of DEPe and DMSO treated astroglia subclusters. D: Subclustered astroglia distribution with DEPe and DMSO treatments.
