# Supplementary material for: Neurotoxicity of diesel exhaust extracts in zebrafish and its implications for neurodegenerative disease
Source: Sci Rep. 2022 Nov 12;12:19371. doi: 10.1038/s41598-022-23485-2 (PMC9653411; doi:10.1038/s41598-022-23485-2)
Supplement: Supplementary file 14 — Supplementary Information 14. [file 41598_2022_23485_MOESM14_ESM.docx]

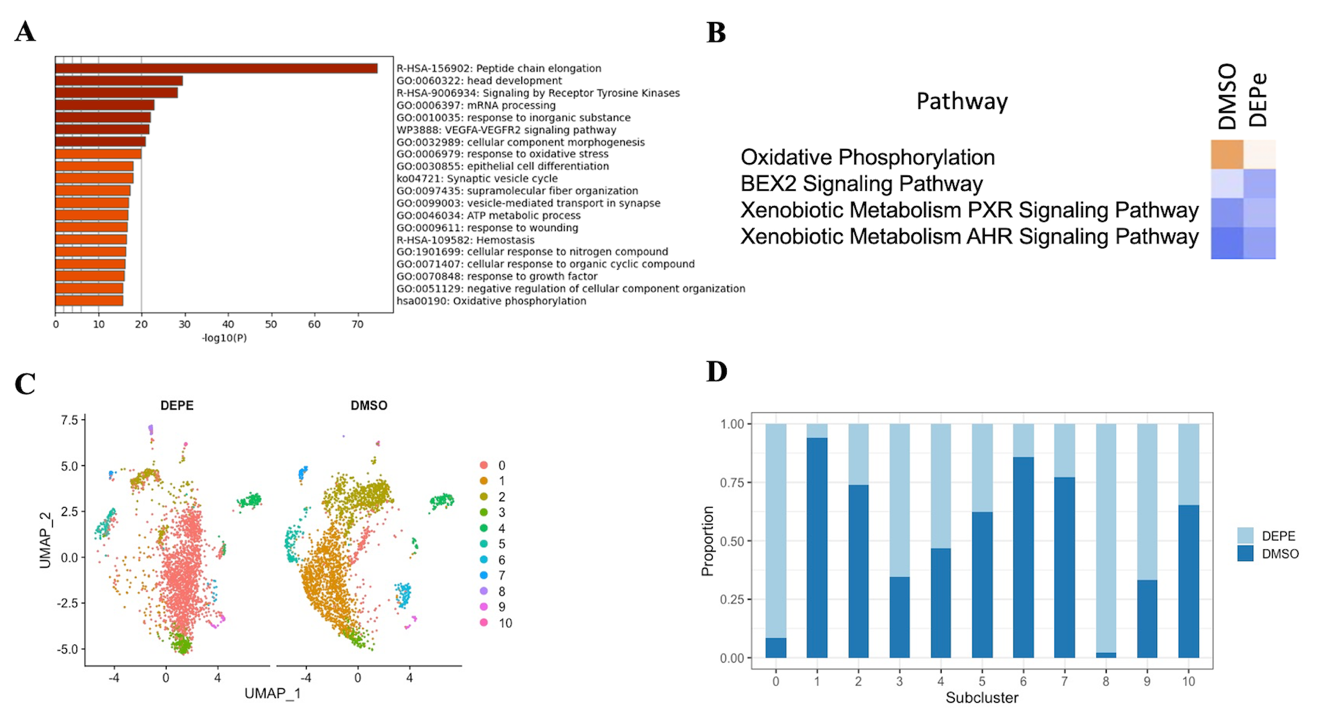


**Supplementary Figure 4:** **Neuronal cluster 1 analysis with and without DEPe exposure**. A: The top enriched biological processes in control neurons. B: Ingenuity Pathways Analysis summary. Darker orange denotes more activated pathways and darker purple denotes less activated pathways (z-score > 2 and z-score < -2 for orange and purple, respectively; p-value < 0.05). C: UMAP plots of DEPe and DMSO treated microglia subclusters. D: Subclustered neuronal distribution with DEPe and DMSO treatments.
