# Supplementary material for: Neurotoxicity of diesel exhaust extracts in zebrafish and its implications for neurodegenerative disease
Source: Sci Rep. 2022 Nov 12;12:19371. doi: 10.1038/s41598-022-23485-2 (PMC9653411; doi:10.1038/s41598-022-23485-2)
Supplement: Supplementary file 15 — Supplementary Information 15. [file 41598_2022_23485_MOESM15_ESM.docx]

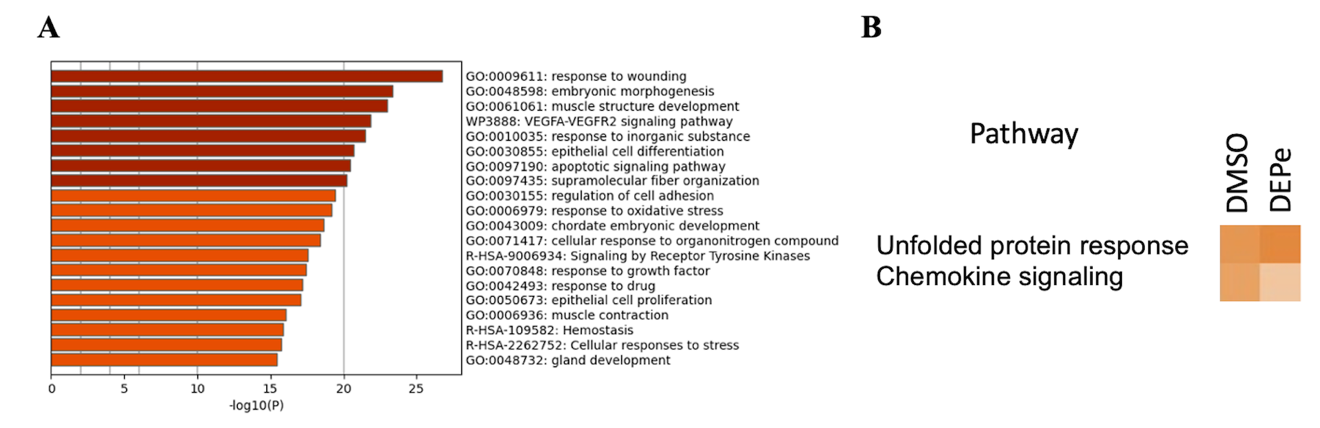


**Supplementary Figure 5: Olfactory Bulb cluster analysis.** A: The top enriched biological processes in the olfactory bulb cluster. B: Ingenuity Pathways Analysis summary. Darker orange denotes more activated pathways (z-score > 2; p-value < 0.05).
